# Increased tumor glycolysis is associated with decreased immune infiltration across human solid tumors

**DOI:** 10.3389/fimmu.2022.880959

**Published:** 2022-11-24

**Authors:** Ivan J. Cohen, Fresia Pareja, Nicholas D. Socci, Ronglai Shen, Ashley S. Doane, Jazmin Schwartz, Raya Khanin, Elizabeth A. Morris, Elizabeth J. Sutton, Ronald G. Blasberg

**Affiliations:** ^1^ Gerstner Sloan Kettering Graduate School of Biomedical Sciences, Memorial Sloan Kettering Cancer Center, New York, NY, United States; ^2^ Department of Pathology, Memorial Sloan Kettering Cancer Center, New York, NY, United States; ^3^ Bioinformatics Core, Memorial Sloan Kettering Cancer Center, New York, NY, United States; ^4^ Department of Epidemiology and Biostatistics, Memorial Sloan Kettering Cancer Center, New York, NY, United States; ^5^ Computational Biology and Medicine Tri-Institutional PhD Program, Weill Cornell Medicine, New York, NY, United States; ^6^ Department of Medical Physics, Memorial Sloan Kettering Cancer Center, New York, NY, United States; ^7^ Department of Radiology, Memorial Sloan Kettering Cancer Center, New York, NY, United States; ^8^ Department of Neurology, Memorial Sloan Kettering Cancer Center, New York, NY, United States; ^9^ Molecular Pharmacology and Chemistry Program, Memorial Sloan Kettering Cancer Center, New York, NY, United States

**Keywords:** tumor metabolism, immunotherapy, tumor microenvironment, solid tumors, glycolysis, immune infiltration

## Abstract

Response to immunotherapy across multiple cancer types is approximately 25%, with some tumor types showing increased response rates compared to others (i.e. response rates in melanoma and non-small cell lung cancer (NSCLC) are typically 30-60%). Patients whose tumors are resistant to immunotherapy often lack high levels of pre-existing inflammation in the tumor microenvironment. Increased tumor glycolysis, acting through glucose deprivation and lactic acid accumulation, has been shown to have pleiotropic immune suppressive effects using *in-vitro* and *in-vivo* models of disease. To determine whether the immune suppressive effect of tumor glycolysis is observed across human solid tumors, we analyzed glycolytic and immune gene expression patterns in multiple solid malignancies. We found that increased expression of a glycolytic signature was associated with decreased immune infiltration and a more aggressive disease across multiple tumor types. Radiologic and pathologic analysis of untreated estrogen receptor (ER)-negative breast cancers corroborated these observations, and demonstrated that protein expression of glycolytic enzymes correlates positively with glucose uptake and negatively with infiltration of CD3^+^ and CD8^+^ lymphocytes. This study reveals an inverse relationship between tumor glycolysis and immune infiltration in a large cohort of multiple solid tumor types.

## Introduction

Immune checkpoint blockade (ICB) with PD1/PDL1-, or CTLA4-blocking antibodies has shown encouraging results, either as monotherapy or in combination with other checkpoint inhibitors or with standard chemotherapies (1, 2). As a monotherapy, some of the best responses were observed in melanoma (objective response rate (ORR) of 45%) (3), PDL1-positive non-small cell lung cancer (NSCLC; ORR 45%) (4–6) and multiple Mismatch-Repair deficient (MMRd) tumor types (ORR of 40-53%) (7–9). The combination of anti-PD1 and anti-CTLA-4 therapies has also shown excellent responses with an ORR in the 40-60% range and long duration of these responses (3, 10–12). ICB also generates improved responses in combination with standard chemotherapies, most notably in lung and breast cancer, with a ~40% increase in 2-year overall survival rates in the immunotherapy-containing arm vs. the chemotherapy-only arm in multiple clinical trials (13–20). Further, multiple studies have recently shown the benefits of neoadjuvant or adjuvant ICB in multiple tumor types (21–27). Since the initial FDA approval of immunotherapy for melanoma and lung cancers, immunotherapies have been cleared by the FDA for various additional tumor types, including head and neck, renal, hepatocellular, colorectal, urothelial, gastric, cervical, breast and Merkel cell carcinomas (28). Moreover, the use of pembrolizumab was recently approved by the FDA in Microsatellite Instability-high (MSI-h) patients, irrespective of the tumor type (29). Although highly encouraging, the majority of patients treated with immunotherapy still fail to respond. This lack of response is likely due in part to the hostile tumor microenvironment (TME) found in solid tumors and its effect on immune infiltrating cells (30).

The Warburg effect describes the preferential utilization of glycolysis in tumor cells even in the presence of oxygen (31). Signaling *via* different oncogenic pathways has been shown to result in increased expression of glycolytic genes with an ensuing increase in glycolytic rates and cell proliferation. Signaling *via* MYC results in the upregulation of various glycolytic genes, such as *LDHA* (32); signaling *via* AKT and BRAF leads to increased glucose uptake in tumor cells (33, 34); and *TP53* inactivation results in increased glycolysis (35). This results in a metabolic tumor microenvironment (mTME) characterized by glucose depletion, lactic acid accumulation and an acidic pH, among other metabolic changes (36–38). Lactic acid is a highly immune-suppressive metabolite that can directly affect many steps involved in mounting a successful anti-tumor immune response (39). Independent studies using mouse models of breast cancer and melanoma have shown that depletion of lactate dehydrogenase A (LDHA) from tumor cells led to a dramatic increase in tumor-infiltrating T-cells and NK cells (40, 41). In addition, activated CD4^+^ and CD8^+^ T cells are highly dependent on glucose (38, 42), whereas regulatory T cells (Tregs) can function effectively in low glucose, high lactate microenvironments. In fact, Tregs have been shown to metabolize lactic acid to fuel their proliferation and support their immune suppressive capacity (43, 44), and inhibition of tumor glycolysis was shown to lead to Treg functional destabilization and increased efficacy of ICB in mouse models of breast cancer and melanoma (45).

Given that one of the best predictors for response to immunotherapy is pre-existing inflammation within tumors (46), we focused on understanding a potential mechanism of immune exclusion that may be important to improve the response to ICB. We hypothesized that increased tumor glycolysis would be associated with decreased immune infiltration across a variety of non-hematologic solid tumor types. Using gene expression profiles from The Cancer Genome Atlas (TCGA) and other independent datasets, we found that increased tumor glycolysis was associated with decreased immune infiltration across multiple cancer types. Our findings may help define not only a subset of patients where ICB is unlikely to be effective, but may also reveal new strategies for the combination of ICB and treatments targeting tumor cell metabolism.

## Materials and methods

### Data processing

Gene expression (RNA) data was downloaded from the National Cancer Institute Genomic Data Commons (NCI GDC) Pan Cancer Atlas Publications website (https://gdc.cancer.gov/about-data/publications/pancanatlas) (47). Clinical data was downloaded from the TCGA-Clinical Data Resource (CDR) Outcome site. We focused our analysis on non-hematologic solid tumor types, and excluded Acute Myeloid Leukemia (LAML), Thymoma (THYM) and Diffuse Large B Cell Lymphoma (DLBC) (n = 30 solid tumor types). Primary and Metastatic tumor samples were included in our study (TCGA Barcode Sample Type Codes 01 and 06). The expression values from the NCI GDC were transformed into log base 2 values.

Gene expression data from the METABRIC (48) study was downloaded from the cBioPortal (https://www.cbioportal.org/datasets) (49); data from GSE65904 (melanoma) and GSE119267 (lung adenocarcinoma) was downloaded from the National Center for Biotechnology Information Gene Expression Omnibus (NCBI GEO). Expression values from GSE65904 were transformed into log base 2 values unless otherwise noted.

EGFR, KRAS and BRAF mutation status were obtained from the cBioPortal for each indicated tumor type. For LUAD, we selected cases with either EGFR L858R mutations or KRAS G12C/V/D/A/S. For SKCM, we selected cases with BRAF V600E mutations. For BRCA, increased androgen receptor (AR) expression cases were counted if they exhibited either (i) high-level amplification of the AR region or (ii) AR mRNA expression >2 standard deviations from the mean relative to all other BRCA cases.

Protein abundance data [as measured by mass spectrometry by the Clinical Proteomics Tumor Analysis Consortium (CPTAC)] for 875 tumor samples across seven cancer types (breast, lung, ovarian, pancreatic, endometrial, brain and colon cancer) was downloaded from the cBioPortal. The Z-score transformed protein abundance values were downloaded and used as is in this study. The GSE140343 lung adenocarcinoma (LUAD) proteomics and clinical data (n=103) was downloaded from Xu, et al. (50) and used as is (only tumor samples were analyzed in our study, not the matching normal tissue samples).

Metabolite abundance data was downloaded from Tang et al. (51) and used as is. ssGSEA T-cell estimates were calculated as above and the relationships between Glucose or Lactate, and different ssGSEA T-cell estimates were plotted.

### Glycolysis and immune signatures

To determine the expression of the glycolysis-related fluorodeoxyglucose (FDG) uptake signature (52), we calculated the Weighted Mean of the genes in this signature for each sample according to the weights in Palaskas, et al. (FDGScore = weightedMean(gene.symbols, gene.weights, na.rm = T) (**Supp Table S1**). This was performed using the log base 2 transformed expression values for each dataset. For genes with more than one probe, the weights of each probe were added as in **Supp Table S1**. To estimate the abundance of T-cell subsets, the single-sample GSEA (ssGSEA) method described by Şenbabaoğlu, et al. was followed [gsva(expression.data, list.of.immune.pathways, method=“ssgsea”)] (53). The expression values without log base 2 were used to estimate immune-cell proportions with ssGSEA. To calculate the enrichment of the 50 Hallmark gene sets from the Molecular Signatures Database (MSigDB) (54), ssGSEA values were calculated as described above for the estimation of T-cell subset abundance, but using the Hallmark gene sets.

To determine the relationships between our signatures (FDGScore, Hallmarks_Glycolysis, and multiple ssGSEA-based T-cell estimates) and clinical parameters (Tumor Stage, Patient Age at Diagnosis, Patient Gender) we used multiple statistical tests. To study the association of our signatures and Tumor Stage, we performed linear regression between our signatures and Tumor Stage, where Tumor Stage was defined numerically from 1 to 4 (Stage I to IV) for TCGA and METABRIC, and defined numerically from 1 to 3 (Primary Tumor, Regional Metastasis, Distant Metastasis) for GSE65904, and we reported the resulting Beta Coefficient (B) and p-value. To study the association of our signatures and Patient Age at Diagnosis (Age), we calculated the Spearman correlation coefficient (rho, r) between our signatures and Age, and we reported the rho and p-value. To study the association between our signatures and Patient Gender, we performed a two-sided t-test between our signatures and Gender, and we reported the male/female expression percentage and p-value.

### Survival analysis

To perform survival analysis on the publicly available datasets (TCGA, METABRIC, GSE65904), patients were stratified into tertiles based on the expression of the different gene signatures (FDGScore, CD8 T Cells) in the tumors. For FDGScore, the log base 2 expression values were used; for CD8 T cells, the raw ssGSEA output was used without any transformation.

The overall survival between patients in the top tertile (“high”) vs. those in the bottom tertile (“low”) was compared using Cox Proportional Hazards Regression analysis with a cutoff of 4,000 days for TCGA and 10 years for METABRIC and GSE65904. Both univariate and multivariate analyses were performed for each gene signature (FDGScore, Hallmarks_Glycolysis, CD8 T cells) and for other available covariates, depending on the study (Age, Gender, Stage, Prior therapies).

For TCGA, the TCGA-Clinical Data Resource (CDR) Outcome file (https://gdc.cancer.gov/about-data/publications/pancanatlas) was used, as per TCGA recommendations (55).

To perform survival analysis on the MSKCC cohort of 49 patients with ER-negative breast cancer, patients were stratified into tertiles based on their expression of LDHA and CD8 by IHC staining. Patients were stratified into the highest LDHA expression tertiles (LDHA H-Score > 180, “LDHA.High”), the highest Mean Glycolysis H Score tertile (Mean Glycolysis H Score > 180, “Gly.High”), the highest CD8 expression tertile (Stromal CD8^+^ % > 20, “CD8.High”), and patients which were not in either of the above top tertiles. The recurrence free survival (RFS) was compared between patients in the Gly.High group vs. non-Gly.High and between CD8.High group vs. non-CD8.High in this cohort using Cox Proportional Hazards Regression and Kaplan-Meier analysis. Data cutoff date for tumor recurrence was November 2 2018.

### Cases

Following institutional review board (IRB) approval (Protocol # 17-236A), cases were retrieved from the Pathology archives of Memorial Sloan Kettering Cancer Center (MSKCC). Patient consents were obtained as described in the protocol, and 49 ER-negative primary breast cancers were reviewed by a pathologist (FP) and classified according to the definitions of the World Health Organization (56). Tumors were graded according to the Nottingham grading system (57). ER and HER2 status were retrieved from the electronic medical records at our institution, and the extent of stromal tumor-infiltrating lymphocytes (sTILs) was evaluated following the recommendation put forward by the International TILs Working Group 2016 (58).

### Immunohistochemistry

Representative formalin-fixed paraffin-embedded whole tissue sections from the 49 ER-negative primary breast cancers were subjected to immunohistochemistry as previously described in the MSKCC Department of Pathology Immunohistochemistry Core Laboratory (59, 60). In brief, sections were incubated for 30 min with the anti-CD3 antibody (Leica Biosystems, Clone LN10) at a 1:200 dilution, anti-CD8 antibody (Dako Omnis, Clone C8/144B) at a 1:100 dilution, anti-LDHA antibody (Cell Signaling, Clone C4B5; #3582) at a 1:300 dilution, or anti-GLUT1 (Polyclonal from AbCam) antibody at a 1:400 dilution. All antibody incubations were followed by a 30 min ER2 pre-treatment (Bond) on a Leica Bond RX platform, followed by Bond Poymer Refine Detection (Leica Biosystems; #DS9800).

Immunohistohemical expression of LDHA and GLUT1 was assessed using the H-score, a semi-quantitative approach based on the sum of individual scores for each intensity (0, negative; 1+, weak; 2+, moderate; 3+, strong) and the percentage of tumor cells displaying a particular expression intensity. The final score is computed with the formula: [1x(%cells 1+) +2 x(%cells 2+) +3x (% cells 3+)] and ranges from 0 to 300. We also computed a composite score of both markers by simply averaging the H-score for GLUT1 and LDHA (Mean Glycolysis H-score).

Immunohistochemical assessment of CD3 and CD8 expression in TILs was recorded as the % of stromal TILs displaying immunoreactivity for these markers. All analyses were performed with observers blinded to the clinical and radiologic features of the cases.

### Imaging

Eighteen of the 49 patients in the MSKCC cohort underwent FDG-PET imaging. The patients ranged in age from 25 – 71 years, and were injected with an average of 431 ± 49 MBq of FDG and imaged at an average of 68 ± 18 min PI on various GE discovery PET scanners (LS,STE, 690, 710) mid-skull to mid-thigh.

Volumetric Regions of Interest (VOIs) were drawn on FDG-PET images over the breast lesion of interest. For each lesion VOI, the maximum and peak standardized uptake values (SUV_max_ and SUV_peak_, respectively) were calculated. The standardized uptake value (SUV) is defined as the tracer uptake in a region divided by the injected activity and patient weight.

SUV_max_ hottest voxel within a defined VOI and SUV_peak_ is calculated by averaging the SUV for all the pixels within a 1 cc sphere containing the lesion VOI such that this average is the largest of all possible such spheres. Both SUV metrics are used to assess the most metabolically active region of a tumor.

## Results

### Inverse correlation between the expression of glycolysis-related genes and immune genes across multiple solid tumor types

Typical response rates to ICB in solid tumor types outside of melanoma, PDL1-positive NSCLC and MMR-deficient tumors is ~5-20% (61–64). Thus, we sought to determine whether increased tumor glycolysis may be associated with decreased immune infiltration. If true, this may (i) allow the identification of patients who may be resistant to ICB; and (ii) reveal tumor glycolysis as a potential target for combination therapies with ICB (44, 45). To initially determine how expression of glycolysis-related genes correlated with tumor immune infiltration, we created a minimal selection of glycolysis genes (one for each of the 10 steps of glycolysis, plus the glucose transporter GLUT1 (*SLC2A1*), lactate dehydrogenase A (*LDHA*) and the lactate transporters *SLC16A1* and *SLC16A3*) and immune genes (consisting of the ‘identity’ genes *CD3, CD4, CD8* and the cytotoxicity genes Granzyme A (*GZMA*) and Perforin 1 (*PRF1*)). This list included glycolysis rate-limiting genes such as *HK2*, *PFKP* and *PKM2* (65, 66). We then performed a preliminary analysis of the expression patterns of these genes in the 30 non-hematologic solid tumor types in the Pan Cancer TCGA cohort (n=9,875). We observed robust co-expression within the glycolysis and immune gene subsets, but minimal inverse correlations between the glycolysis and immune genes, with the strongest negative correlation observed between *GPI* and *CD4*: r = -0.08, p = 2.16e^-15^) (**Supp Figures S1A, B**).

**Figure 1:**
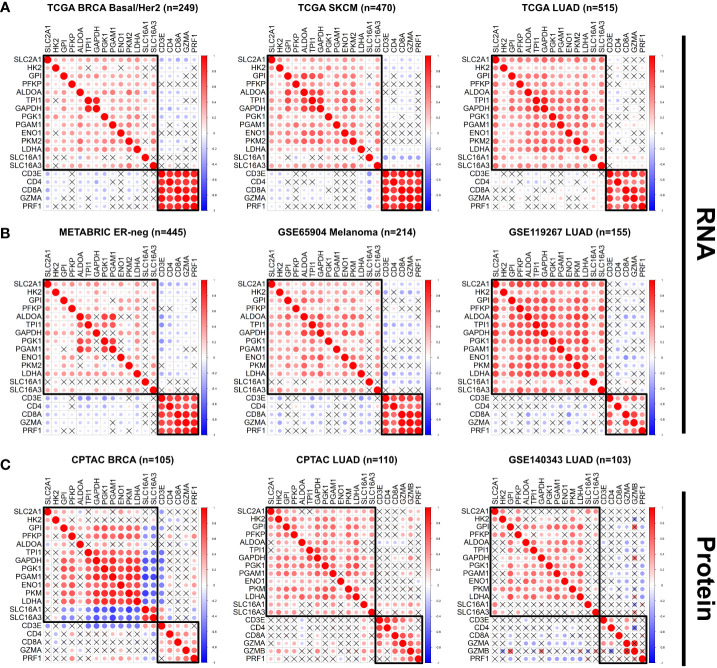
The expression of glycolysis- and immune-related genes is negatively correlated across multiple solid tumor types. **(A, B)** The correlation between expression of selected glycolysis and immune genes was plotted for individual tumor types in the TCGA dataset **(A)** Basal/Her2 Breast Cancer (BRCA), Skin Cutaneous Melanoma (SKCM), Lung Adenocarcinoma (LUAD), and in the independent datasets **(B)** ER-negative METABRIC, GSE65904 Melanoma, GSE119267 LUAD). **(C)** The correlation between protein abundance of specific glycolysis and immune proteins was plotted for specific tumor types in the CPTAC cohort (BRCA, LUAD) and the GSE140343 LUAD cohort. Red = positive correlation; blue = negative correlation. The size and intensity of the circles are proportional to the Pearson r coefficient. Pearson correlation coefficients that were not statistically significant (p>0.05) are marked with an X.

When divided into individual cancer types and subtypes, however, there were strong inverse correlation patterns between specific glycolysis and immune genes, especially in the Basal and Her2 subtypes of breast cancer (BRCA) (with the strongest negative correlations occurring between *SLC2A1* and *CD8A*: r = -0.32, p = 1.72e^-7^), skin cutaneous melanoma (SKCM) (*SLC16A1* vs. *CD3E*: r = -0.42, p = 9.83e^-22^), and lung adenocarcinoma (LUAD) (*TPI1* vs. *CD4*: r = -0.22, p = 4.66e^-7^) (**Figure 1A**; **Supp Figure S1B**). We sought to validate these findings in independent datasets, including the METABRIC breast cancer cohort (23); the GSE65904 dataset [comprised of 214 melanoma samples (67)], and a cohort of 155 LUAD samples (GSE119267) for which gene expression profiles were publicly available (68). We observed similar expression patterns in these datasets, wherein some glycolytic genes showed strong and significant inverse correlations with specific immune genes in specific tumor types (with the strongest negative correlations occurring in ER-negative breast cancer from METABRIC: *TPI1* vs. *CD3E*: r = -0.44, p = 2.39e^-22^; GSE65904 Melanoma: *LDHA* vs. *CD3E*: r = -0.43, p = 6.03e^-11^; GSE119267 *LUAD*: *ENO1* vs. *CD8A*: r = -0.43, p = 2.57e^-8^) (**Figure 1B**; **Supp Figures S1A, B**). Additionally, the expression of the glycolysis rate-limiting genes *HK2, PFKP* and *PKM2* showed expression patterns similar to non-rate-limiting genes in the Pan Cancer TCGA cohort. The correlation between the rate-limiting genes *HK2* vs. *CD4* was r = -0.03, p = 0.01; *PFKP* vs. *CD4*: r = 0.05, p = 3.43e^-6^; *PKM2* vs. *CD4*: r = 0.09, p = 6.14e^-21^). Similarly, the correlation between specific non-rate limiting glycolysis genes was *GAPDH* vs. *CD4*: r = 0.005, p = 0.61, *ALDOA* vs. *CD4*: r = -0.02, p = 0.02 (**Supp Figure S1A**).

We then delved deeper into the molecular subtypes of breast cancer, lung adenocarcinoma and melanoma. We found that in contrast to breast cancer samples from the ER-negative and Basal/Her2 subtypes, samples from the ER-positive/LumA-LumB subtypes showed weaker negative correlations (*TCGA BRCA LumA/LumB*: *ALDOA* vs. *CD8A*: r = -0.22, p = 2.0e^-9^; *METABRIC ER-positive*: *ALDOA* vs. *CD3E*: r = -0.24, p = 1.24e^-20^) (**Supp Figures S1A, B**). Additionally, a cohort of breast cancer patients with increased expression of the androgen receptor gene (*AR*) showed a paucity of statistically significant negative correlations between glycolysis and immune genes (**Supp Figure S2A**). Within the SKCM cohort, we found that tumors with a BRAF V600E mutation lost the strong negative correlations observed in the *BRAF* WT cohort between multiple glycolytic genes and immune genes, with the exception of *LDHA* and *SLC16A1* (strongest negative correlation between *SLC16A1* vs. *CD3E*: r = -0.34, p = 9.68e^-6^) (**Supp Figure S2B**). Similar results were observed in LUAD tumors with either *EGFR* L858R or *KRAS* G12 mutations, wherein a significant portion of the negative associations between glycolysis and immune genes lost statistical significance (no significant negative correlations observed in the *EGFR* L858R cohort; strongest negative correlation in the *KRAS* G12 cohort: *TPI1* vs. *CD4*: r = -0.018, p = 0.038) (**Supp Figures S2C, D**).

We performed similar analyses on available proteomics datasets from (i) the Clinical Proteomic Tumor Analysis Consortium (CPTAC) (69–76) and (ii) a LUAD cohort of 103 tumor samples (GSE140343) (50). We again found strong negative correlations in the protein abundance of specific glycolysis and immune genes in the CPTAC BRCA (GAPDH vs. CD3E: r = -0.58, p = 8.38e^-8^) and CPTAC LUAD (ALDOA vs. CD3D: r = -0.26, p = 0.019) cohorts, and in the GSE140343 LUAD cohort (LDHA vs. PRF1: r = -0.41, p = 0.003) (**Figure 1C**). We also found significant negative correlations in the PAAD (ENO1 vs. CD3E: r = -0.46, p = 4.030e^-7^), UCEC (TPI1 vs. GZMB: r = -0.31, p = 0.015), GBM (PFKP vs. CD3E: r = 0.50, p = 7.42e^-3^), OVCA (GPI vs. CD4: r = -0.28, p = 9.69e^-3^) and LUSC (SLC2A1 vs. CD3E: r = -0.44, p = 2.52e^-6^) cohorts, but minimal negative correlations in the CPTAC COAD (HK2 vs. CD8A: r = -0.35, p = 0.02) cohort (**Supp Figure S3A**). Moreover, our analyses revealed a robust negative correlation between LDHA protein abundance and the extent of immune-cell and CD8^+^ T cell infiltration in the GSE140343 LUAD proteomics cohort, where the negative associations between LDHA and immune cell infiltration were stronger in the EGFR wildtype (WT) cases compared to the EGFR mutant cases (**Supp Figures S3B, C**). Thus, our preliminary analysis on both mRNA and protein datasets (TCGA, METABRIC, GSE65904, GSE119267, CPTAC BRCA, PAAD, UCEC, GBM, OVCA, LUSC and GSE140343) suggest that increased tumor glycolysis may lead to decreased immune infiltration across multiple solid tumor types.

### Increased expression of a glycolysis signature is associated with depletion of CD8^+^ T-cells in most solid tumor types

To quantify the expression patterns of glycolysis and immune related genes, we applied a previously developed signature that predicts fluorodeoxyglucose (FDG) uptake in patients and in cell lines (52). This signature, referred to as FDGScore in our study, has the advantage of having been developed by assessing FDG uptake both in patients (ensuring clinical relevance) and cell lines *in-vitro*, ensuring that the signature takes into account uptake and retention of the radiotracer without confounding factors found in purely clinical data sets, such as tumor size, heterogeneity, vessel quantity, and radiotracer delivery. In addition, to estimate the proportion of different immune cell types within tumors from TCGA as well as other datasets, we implemented the single-sample GSEA (ssGSEA) method (53, 77). This method has the advantage of (i) producing near-Gaussian curves of the immune estimates; and (ii) ease of implementation into independent datasets.

We first characterized the expression patterns of FDGScore in our cohorts, and found that increased FDGScore expression was significantly associated with Tumor Stage across the entire Pan Cancer TCGA cohort (Regression Beta Coefficient (B) = 0.16, p = 6.65e-^42^) and the METABRIC cohort (B = 0.29, p = 1.03e-^95^), but not in the GSE65904 Melanoma cohort (B = 0.05, p = 0.398) (**Supp Figures S4A–C**). When analyzed in individual tumor types, we found that 8/30 (27%) of tumor types showed a statistically significant association between FDGScore and Tumor Stage (**Supp Table S2**). Further, we observed minimal differences in FDGScore expression with increasing Age or Male vs. Female Gender, both at the PanCancer level and within individual tumor types (**Supp Figures S4A–C** and **Supp Table S2**).

**Figure 2:**
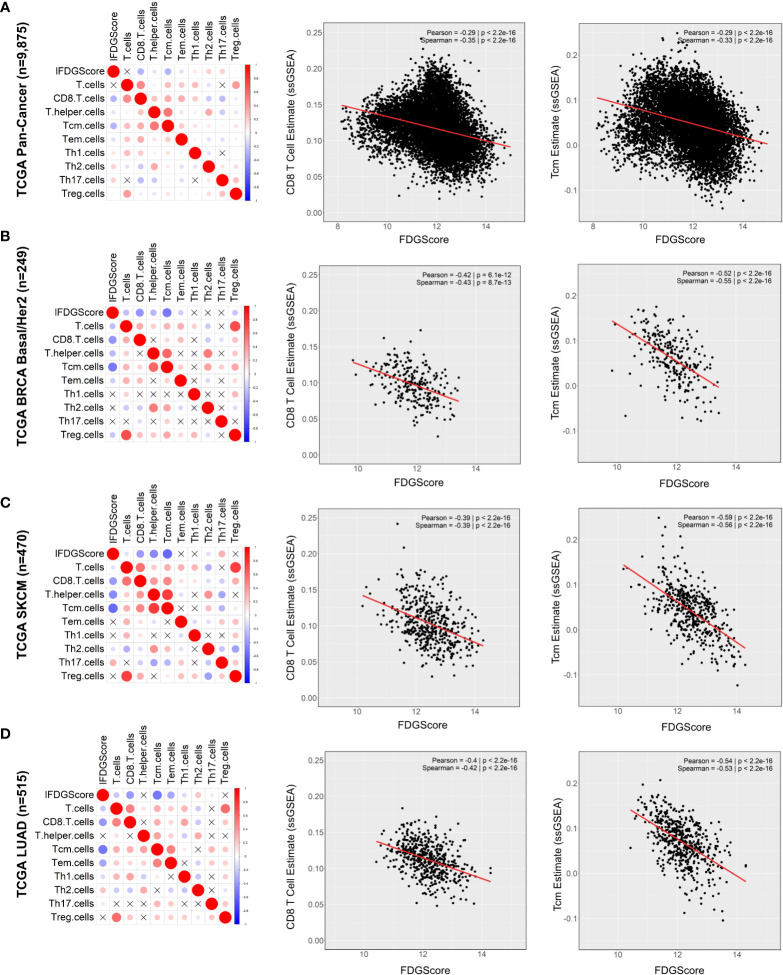
Expression of the glycolysis signature FDGScore is inversely correlated with multiple estimates of T cell infiltration across solid tumors. **(A–D)** The correlation profiles of the FDG uptake signature (FDGScore) and the estimates of T cell subset abundance (as measured by ssGSEA) were calculated and plotted for the entire TCGA Pan Cancer cohort **(A)** and for individual tumor types within TCGA **(B–D)** (left). The expression of the FDGScore vs. CD8 (middle) and Tcm (right) T cell estimates is also shown with the calculated Pearson and Spearman coefficients. Red = positive correlation; blue = negative correlation. The size and intensity of the circles are proportional to the Pearson r coefficient. Pearson correlation coefficients that were not statistically significant (p>0.05) are marked with an X.

We then sought to determine the correlation between the estimate of the proportion of all the T-cell subsets (by the ssGSEA method) and our FDG signature (FDGScore) across the entire TCGA cohort (n=9,875). We found that FDGScore was most negatively correlated with the CD8 T cell estimate (Pearson rho = -0.29, p < 1.42e^-186^) and the central memory T cell estimate (T_cm_; r = -0.29 and p < 3.02e^-192^) (**Figure 2A**). When conducting the analyses in individual tumor types we found that FDGScore was significantly negatively correlated with the CD8 T cell estimate in 23/30 tumor types tested (Pearson r range: -0.57 - -0.09). Similarly, FDGScore was negatively correlated with the Tcm estimate across all cancer types in a statistically significant manner with the exception of CHOL (Pearson r range: -0.72 – 0.19) (**Supp Table 3**).

We then focused on BRCA, SKCM and LUAD and found that they were among the top 10 tumor types with the strongest negative correlations between FDGScore expression and the CD8 T cell estimate (*BRCA Basal/Her2*: r = -0.42, p = 6.15e^-12^; *SKCM*: r = -0.39, p = 1.21e^-18^; *LUAD*: r = -0.40, p < 2.36e^-21^) and the T_cm_ estimate (*BRCA Basal/Her2*: r = -0.52, p = 6.35e-19; *SKCM*: r = -0.59, p = 3.96e^-45^; *LUAD*: r = -0.54, p < 1.75e^-39^) (**Figures 2B–D** and **Supp Table 3**). These observations extended to our independent datasets, with negative associations found between FDGScore and CD8 T cells (*METABRIC ER-negative*: r = -0.42, p < 2.69e^-20^; *GSE65904 (Melanoma)*: r = -0.34, p = 3.67e^-7^; *GSE119267 (LUAD)*: r = -0.44, p = 7.02e^-09^) and T_cm_ cells (*METABRIC ER-negative*: r = -0.26, p < 2.58e^-8^; *GSE65904 (Melanoma)*: r = -0.30, p = 7.17e^-6^; *GSE119267 (LUAD)*: r = -0.40, p = 2.13e^-7^) (**Supp Figures S5A–C**). Further, within breast cancer subtypes, we found that the LumA/LumB subtype of BRCA in TCGA, and the ER-positive subtype in METABRIC had weaker negative correlations between FDGScore and immune cell infiltration, although statistical significance was maintained (**Supp Figures S6A**). These results support our hypothesis that increased tumor glycolysis may create a microenvironment that is hostile to infiltrating T-cells, especially CD8^+^ T-cells and T_cm_ cells.

We then sought to validate the findings obtained with the FDGScore signature using a different glycolysis gene signature. We chose to focus on the Molecular Signatures Database “Hallmark” Gene Set Collection (54). From this collection of 50 “Hallmark” gene sets, we selected the “Glycolysis” gene set and quantified its associations with FDGScore and the various ssGSEA-derived T-cell estimates, as above. We observed a strong positive correlation between HM_Glycolysis and FDGScore across all tumor types we tested (Pearson r range = 0.40-0.78). (**Supp Table 3**). In accordance with our FDGScore-based findings, we observed a robust negative correlation between the Hallmark Glycolysis gene signature (referred to as HM_Glycolysis in our study) and T cell estimates across multiple solid tumor types. Across the entire TCGA cohort, HM_Glycolysis was strongly negatively correlated with the CD8 T cell estimate (Pearson r = -0.40, p < 2.2e-200) and the T_cm_ estimate (r = -0.65, p = 4.18e-284) (**Supp Figure S7A** and **Supp Table 3**). As above, we quantified these relationships within individual tumor types and found that 27/30 solid tumor types in the TCGA dataset showed a statistically significant negative association between HM_Glycolysis and the CD8 T cell estimate (**Supp Table 3**). In addition, similar to our observations with FDGScore, we observed robust negative associations between HM_Glycolysis and the CD8 and T_cm_ estimates in the TCGA BRCA Basal/Her2, SKCM and LUAD cohorts (**Supp Figures S7B–D**). We also observed negative associations in the METABRIC ER-negative, GSE65904 and GSE119273 cohorts (**Supp Figures S8A–C**), and weaker negative correlations in the TCGA BRCA LumA/LumB and METABRIC ER-positive cohorts (**Supp Figures S9A, B**). Thus, using a different glycolysis signature from the Broad MSigDB, we validated our initial findings and showed that increased expression of a different glycolysis signature is strongly and significantly associated with decreased expression of multiple T-cell estimates across most solid tumor types we studied.

Additionally, to investigate whether the abundance of lactate itself was associated with the levels of T cell infiltration in human tumors, we leveraged the metabolomics dataset published by Tang et al. (51). The authors collected a cohort of 23 breast tumors that were fully characterized by TCGA (15/23 cases being LumA/LumB subtypes, 8/23 being Basal/Her2 subtypes), and they further analyzed the metabolome of these tumors by gas-chromatography/mass spectroscopy (GC/MS) and liquid-chromatography/mass spectroscopy (LC/MS), which included both glucose and lactate. We found that FDGScore was negatively correlated with glucose levels (r = -0.52, p = 0.012) and positively correlated with lactate levels (r = 0.49, p = 0.017) (**Supp Figures S10A, B**). We further observed that lactate levels were negatively correlated with multiple ssGSEA-based T cell estimates (lactate vs. Tcm: r = -0.45, p = 0029; lactate vs. T Helper: r = -0.44, p = 0.037; lactate vs. CD8: r = -0.31, p = 0.15) (**Supp Figures S10A, C**). Taken together, in addition to the transcriptomic and proteomic data presented above, the analysis of a metabolomic dataset lends further support to the notion that increased levels of glycolysis and lactate accumulation are associated with decreased immune infiltration in human breast tumors.

### Expression of FDGScore and CD8 T-cell signatures is associated with prognosis

**Figure 3:**
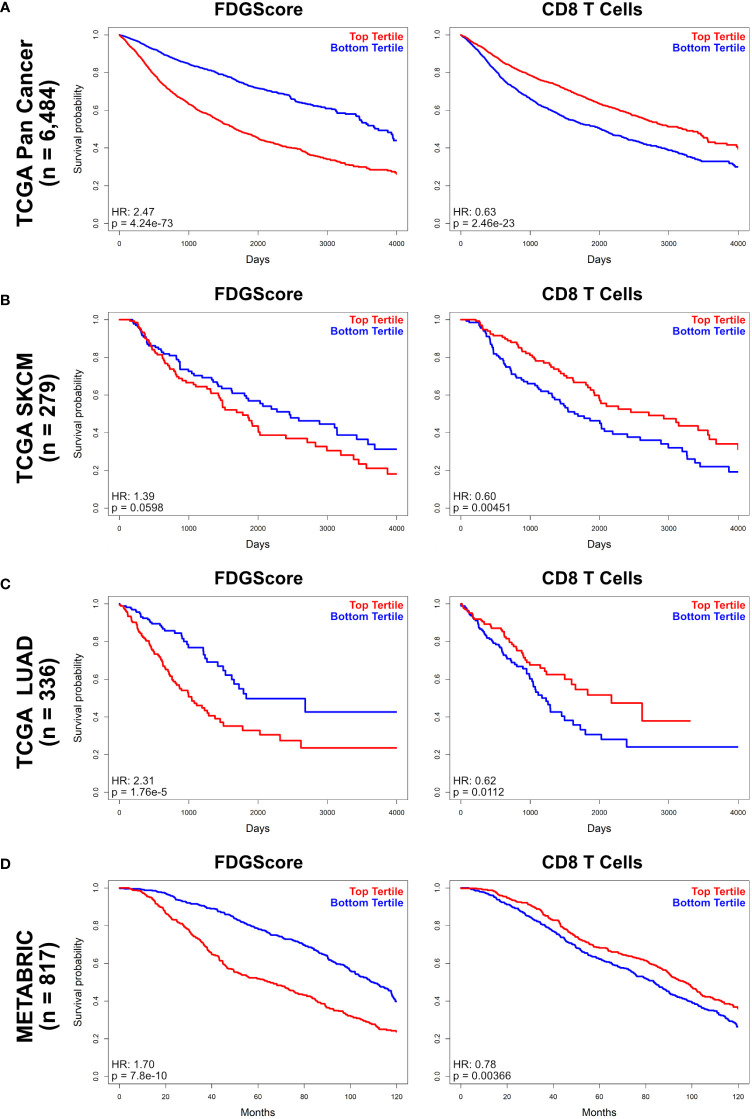
Overall survival by FDGScore and the CD8 T cell estimate in solid tumors. **(A–D)** Kaplan-Meier survival analysis was performed for the entire TCGA cohort **(A)**, as well as individually for the TCGA SKCM **(B)**, TCGA LUAD **(C)** and METABRIC **(D)** cohorts. The disease-specific survival probability of patients was measured in the top tertile vs the bottom tertile of expression of either FDGScore (*left*) or CD8 T cell estimate (*right*) for each cancer type, and the Hazard Ratio (HR) was calculated.

We next sought to determine whether FDGScore and the CD8-T cell ssGSEA estimate correlate with patient survival. Our analyses in all patients of the Pan Cancer TCGA cohort revealed that high FDGScore expression was associated with poor prognosis (HR = 2.47, 95% CI = 2.24-2.72, p = 4.25e^-73^) whereas CD8 T cell estimates was associated with improved prognosis (HR = 0.63, 95% CI = 0.58-0.69, p = 2.46e^-23^) in univariate analysis (**Figure 3A**; **Supp Table S4**). We also found that a high FDGScore expression was associated with poor prognosis in specific individual tumor types (*METABRIC HR*: 1.70, 95% CI = 1.43-2.01, p = 7.^18e-10^; *TCGA SKCM HR*: 1.39, 95% CI = 0.98-1.97, p = 0.0598; *TCGA LUAD HR*: 2.31, 95% CI = 1.58-3.39, p = 1.76e^-5^), while a high CD8 T-cell signature was consistently associated with improved prognosis (*METABRIC HR*: 0.78, 95% CI = 0.66-0.92, p = 3.66e^-3^; *TCGA SKCM HR*: 0.60, 95% CI = 0.43-0.86, p = 4.51e^-3^; *TCGA LUAD HR*: 0.62, 95% CI = 0.43-0.90, p = 1.12e^-2^) (**Figures 3B–D**; **Supp Table S4**). In contrast to the stronger negative correlations found in the Basal/Her2/ER-negative cohorts of the TCGA BRCA and METABRIC cohorts compared to the Luminal/ER-positive cohorts, we found minimal differences in prognosis between Basal/Her2/ER-negative and Luminal/ER-positive breast cancer cohorts (**Supp Figures S11A-D**). Additionally, we again sought to validate our findings using the HM_Glycolysis signature and similarly found that increased HM_Glycolysis expression was associated with poor prognosis both in the entire Pan Cancer TCGA cohort as well as within the individual tumor types that we studied (**Supp Figures S12A-D**).

Further, FDGScore was independently associated with poor prognosis in the entire Pan Cancer TCGA cohort in multivariate analysis (HR = 2.47, 95% CI = 2.24-2.72, p = 4.25e^-73^), while the CD8 T cell estimate was associated with improved prognosis (HR = 0.69, 95% CI = 0.61-0.77, p = 3.20e^-10^) (**Supp Table S5**). Similarly, HM_Glycolysis was also independently associated with poor prognosis in the Pan Cancer TCGA cohort (HR = 1.62, p = 3.08e^-25^) (**Supp Table S5**). When analyzed within individual tumor types, many of the associations with prognosis remained significant (FDGScore remained significantly associated with prognosis in the TCGA LUAD, METABRIC and GSE65904 cohorts; while the CD8 T cell estimate remained significantly associated with improved prognosis in the TCGA LUAD and SKCM cohorts; **Supp Table S5**). These data suggest that increased expression of glycolytic genes is significantly associated with poor prognosis, while increased expression of the CD8 T-cell signature is modestly and significantly associated with improved prognosis across multiple tumor types.

### Increased protein expression of glycolytic enzymes is associated with decreased immune infiltration in primary ER-negative breast tumors

ER-negative breast cancer was found to display significant negative correlations between glycolysis and immune infiltration by transcriptomic and proteomic profiling (**Figures 1**, **2**). To corroborate these observations, we assessed the protein expression levels of surrogate markers of glycolytic activity and immune infiltration in 49 treatment-naïve, primary breast cancers, including 39 triple-negative breast cancers (TNBC; i.e., ER-negative, PR-negative and HER2-negative) and 10 ER-negative/HER2-positive breast cancers using IHC staining (**Supp Table S6**). The median age of the patients was 47 years old (range: 25-71) and the median size of the tumors was 2.4 cm (range: 0.9 – 5 cm). Fifty-one percent (25/49) and 45% (22/49) of tumors were of T1 and T2 stage, respectively, whilst one tumor was of T3 and another one T4 (1/49; 2% each). Fifty-six percent (27/48) of patients were node positive, and 18/49 of patients had undergone an FDG PET scan prior to therapy or surgery.

**Figure 4:**
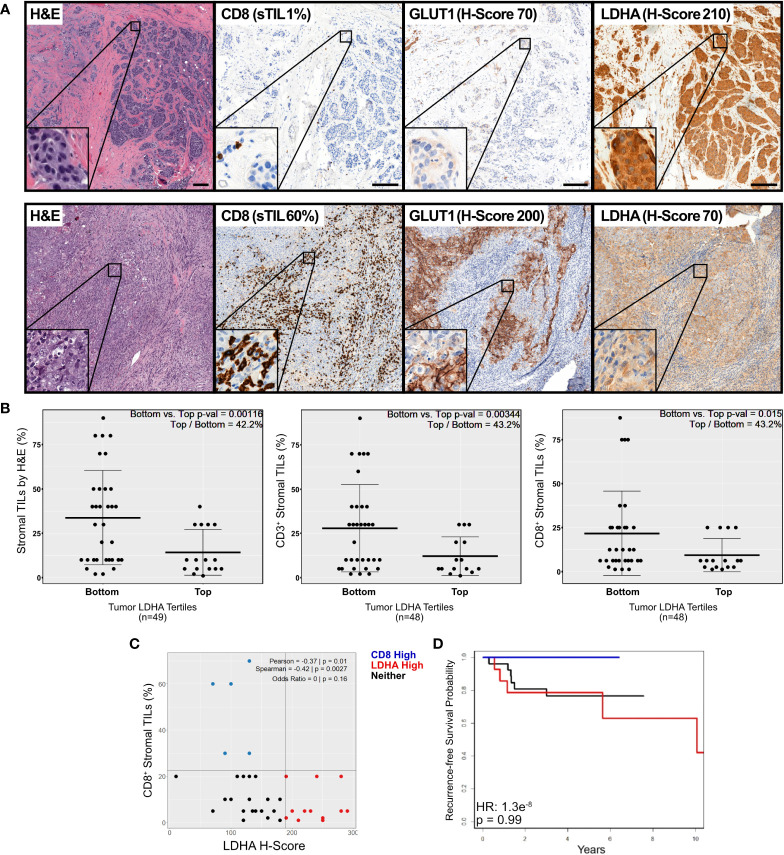
IHC staining of primary breast tumor samples reveals an inverse association between expression of glycolytic and immune markers. **(A)** Representative micrographs of immunohistochemical staining for CD8, GLUT1 and LDHA in our cohort of 49 primary, untreated ER-negative breast tumor samples. Shown are selected sections of tumors with high LDHA expression and low stromal CD8^+^ T-cell infiltrate (*top*), and with low LDHA expression and high stromal CD8^+^ T-cell infiltration (*bottom*). **(B)** The extent of stromal lymphocytic infiltration (sTILs) was quantified (by H&E staining, *left*; or by IHC staining of CD3^+^ (*middle*) or CD8^+^ (*right*) T cells) and plotted in tumors in the top tertile of LDHA expression vs. tumors in the bottom 2 tertiles of LDHA expression, as measured by the H-Score. **(C)** The percentage of stromal CD8^+^ TILs was plotted against the LDHA H-Score, and data was color coded according to whether the sample was in CD8 High (*blue*), LDHA High (*red*) or Neither (*black*) group. The Odds Ratio for CD8 High and LDHA High was calculated and displayed. **(D)** Recurrence-free survival was calculated and Kaplan-Meier plots were plotted for all tumors according to their phenotype as described in **(C)**.

Our analysis revealed a strong positive correlation between FDG uptake and GLUT1 expression (Pearson r = 0.67; p = 0.002) (**Figure 4A**) that was further enhanced in the Mean Glycolysis H-Score (see Methods) (Pearson r = 0.70; p = 0.001) (**Supp Figure S13A**). These results suggest that the expression of glycolytic markers can be used as an indicator of glycolytic activity in breast tumors. We then quantified the relationship between FDG uptake and immune-cell infiltration and found no significant associations between FDG uptake and either total stromal TILs, CD3^+^ or CD8^+^ TILs (**Supp Figure S13B**). We also studied how expression of glycolytic enzymes and immune infiltration affects patient recurrence-free survival (‘RFS’). Notably, we found that increased expression of glycolytic markers was associated with poor prognosis (HR 3.44, p = 0.0529), whereas a numerical association between stromal immune infiltration and longer RFS (HR = 1.2e^-8^, p = 0.99) was observed, although this analysis did not reach statistical significance, likely due to the small sample size and number of events (**Supp Figure S13C**).

Next, we sought to determine the relationship between the expression of the glycolytic enzyme LDHA and the extent of lymphocytic infiltration. We separated our samples into either the top tertile of LDHA expression vs. the bottom 2 tertiles of LDHA expression. We found that tumors with the highest levels of tumor-cell LDHA expression displayed a significantly reduced infiltration of stromal TILs (*left*), and of CD3^+^ (*middle*) and CD8^+^ (*right*) lymphocytes (**Figure 4B**; p = 0.001, 0.003 and 0.015, respectively). Moreover, the extent of CD8^+^ stromal tumor-infiltrating lymphocytes (sTILs) inversely correlated with the LDHA expression when used as a continuous variable (Pearson rho = -0.37, p = 0.01) (**Figure 4C**). Given that the association between CD8 sTIL % and LDHA H Score was not linear, we also calculated the odds of a tumor having both high CD8 sTIL % and high LDHA H Score, and found that the probability for a tumor to be in the top tertile for both was 0, although this test did not reach statistical significance (p = 0.16), likely due to low n. Further, three clusters with different extents of CD8-positive sTILs and LDHA expression levels were identified (LDHA.High, CD8.High, or Neither). We sought to determine whether patients in these three clusters would have differences in their recurrence-free survival. Our analysis revealed that patients in the CD8.High group (with high levels of CD8^+^ stromal TILs and low LDHA), tended to have a better recurrence-free survival than those in the remaining two clusters (**Figure 4D**). Although statistical significance was not achieved (due to a low “n”), no patients in cluster 3 had a recurrence event as of data cutoff. In contrast, patients in the LDHA.High or Neither groups had high and moderate LDHA levels and low CD8^+^ TILs, respectively, and 20-40% of patients in these clusters experienced tumor recurrence. Taken together, these findings show that increased metabolic tumor activity is associated with immune exclusion and poor prognosis.

## Discussion

Numerous published studies have demonstrated a direct and strong inhibitory effect of tumor glycolysis and lactic acid on immune cell function, mostly using *in-vitro* and *in-vivo* models of disease (41, 42, 78, 79). Given the robust effect observed in these studies, we hypothesized that this immune suppressive effect of tumor glycolysis may be widespread and would also be observed in patients, across multiple solid tumor types. Indeed, we demonstrate that in most solid tumor types in the TCGA dataset, as well as in select independent datasets, there is a strong negative correlation between expression of two glycolysis signatures and CD8 and memory T-cell infiltration.

The Warburg effect, discovered in the 1920’s, is a common finding across multiple cancer types (80). Multiple lines of evidence suggest that the Warburg effect, in addition to being important for providing the metabolic building blocks for rapid cell proliferation (81), is highly immune suppressive. The depletion of glucose and the concomitant accumulation of lactic acid has been shown to directly affect multiple immune cell types, inhibiting anti-tumor immune cells while promoting the formation, survival and function of pro-tumorigenic immune cells. For example, two recent studies have elegantly shown that Tregs become destabilized and lose their immune-suppressive potential with increased glucose uptake and increased glycolytic rates that may be found in tumors with decreased tumor glycolysis (44, 45). In contrast, Tregs with decreased glucose uptake show increased uptake of extracellular lactate and increased immune suppressive potential. Further, recent studies have also shown that glycolytic metabolites can directly regulate the nutrient-sensing PI3K/mTOR pathway (82–84), and that glycolysis and lactic acid can directly affect gene expression by promoting histone acetylation and lactylation (85, 86), expanding the tumor-promoting effects of the Warburg effect. In this study, we propose that glycolysis-induced local immune suppression in solid tumors is yet another critical contribution of the Warburg effect to tumor progression, and this may help explain why the Warburg effect is central to tumorigenesis across multiple tumor types.

We initially showed that expression of specific glycolysis-related genes is negatively correlated with expression of immune-related genes across multiple tumor types, both at the mRNA and protein level. In our study, we did not observe differences in the expression pattern of the glycolysis rate-limiting genes *HK2, PFKP* and *PKM2* when compared to the expression of non-rate limiting steps of glycolysis. We expanded our observations by studying the relationship between established glycolysis (52) and immune (53) signatures. We found a strong inverse relationship between the glycolysis and the CD8 T-cell signatures in 23/30 solid tumor types in the TCGA cohort (**Supp Table 3**). We further observed that the Central Memory T-cell signature was significantly negatively correlated with expression of the FDGScore (**Supp Table 3**). This suggested that increased glycolysis may not only blunt CD8 T-cell infiltration, but it may also negatively affect the phenotype of T_cm_ T-cells, another critical component of the anti-tumor immune response. We validated these findings using the well-established “Hallmarks” gene signatures from the MSigDB, and found similar negative associations between HM_Glycolysis and the CD8 and T_cm_ estimates (**Supp Table 3**). Additionally, we leveraged the metabolomic dataset from Tang et al. (51) to show that the levels of lactate itself were negatively associated with T cell infiltration (**Supp Figure S9**). We corroborated these findings by analyzing 49 primary and treatment-naïve breast tumor samples, where we observed a strong inverse relationship between the expression of glycolysis markers (GLUT1 and LDHA) and stromal infiltration of CD3^+^ and CD8^+^ T-cells (**Figure 4**). We found that expression of GLUT1 and LDHA correlated strongly with ^18^F-FDG uptake as measured by PET (**Supp Figure S13A**). Further, in agreement with the transcriptomic analyses performed here, there was a strong negative correlation between expression of LDHA protein and all 3 immune variables (CD3^+^, CD8^+^ and total lymphocyte counts, **Figure 4B**). Thus, we consistently show a strong inverse relationship between expression of glycolytic and immune markers across multiple solid tumor types.

A potential caveat of our approach is that we studied the relationships between just two glycolysis signatures and a single method for estimating immune cell abundance (ssGSEA). However, while numerous other glycolysis-related signatures have been described (87–95), these signatures are (i) mostly composed of genes that are not directly involved in glycolysis (such as *COL5A1, HMMR, STC1*, among others); and (ii) have been developed by their association with prognosis/survival rather than with the metabolic activity of tumors. We initially chose the glycolysis signature described by Palaskas, et al. (52) given that (i) this signature was developed by the direct measurement of FDG uptake in cell lines and in patients; and (ii) this signature is composed solely by genes involved in glucose metabolism. Additionally, to validate our findings, we chose the Hallmarks-Glycolysis gene signature as it was developed by the Molecular Signatures Database (MSigDB). The Hallmark gene lists were created using a combination of bioinformatic approaches and expert curation that led to Hallmark gene sets with reduced variation and redundancy while attaining increased coherent expression within each Hallmark gene list (54). The HM_Glycolysis signature showed highly concordant expression with FDGScore across tumor types, and showed negative associations with various T-cell estimates to a similar degree as we observed when using FDGScore (**Supp Table 3**).

Currently, there are multiple methods for estimating immune-cell abundance (96). We chose the ssGSEA approach taken by Senbabaoglu, et al. (53) given that (i) it produces normally distributed scores for multiple immune cell types, making downstream statistical analyses more straightforward; and (ii) ease of implementation to independent datasets, as demonstrated in previous studies that used ssGSEA for immune deconvolution of various solid tumors (77, 97). However, although our mRNA analysis may be limited to individual gene signatures, these findings are consistent with the proteomic analysis by the CPTAC described in **Figure 1** and **Supp Figure S3**, and with our IHC analysis of human breast tumor tissues described in **Figure 4**. Although our mRNA studies are based on a number of different cohorts and encompass >10,000 patient samples, a limitation of our study is that the IHC findings are based on a limited number of patients (49 breast cancer patients). Thus, further studies to confirm our observations using protein-based methods in diverse tumor types are warranted. Another caveat of our study is that while we show strong negative associations between glycolysis and immune-cell infiltration, the prognostic value of the FDGScore and T-cell signatures, and IHC staining of glycolytic and immune markers, although statistically significant in many cases, is not universally strong and statistically significant. Although the associations between our glycolysis and immune gene signatures showed robust associations with overall survival in the Pan Cancer TCGA cohort of 30 solid tumor types (**Figure 3A**), the association between our gene signatures and prognosis were modest when analyzed within individual tumor types (**Figures 3B, C** and **Supp Tables S4, S5**). We speculate that the robust associations in the Pan Cancer cohort may arise from increased variability in gene signature expression and prognosis between the 30 solid tumor types studied, while the modest associations observed within individual tumor types may arise from decreased variability within individual disease types.

We note that a number of studies have quantified the relationship between FDG uptake and TIL abundance in solid tumors, with some studies showing a positive (albeit small) correlation between FDG uptake and TIL counts (98, 99). In our study, we did not observe a significant correlation between FDG uptake and TIL counts in our cohort of 49 breast cancer patients (**Supp Figure S13B**). In contrast, throughout our study we have shown a significantly negative association between tumor glycolysis and immune infiltration. These contrasting results could be explained by the fact that while the FDGScore signature is indeed *associated* with FDG uptake, it is mainly composed of glycolysis and glucose metabolism genes. As such, FDGScore expression in tumors should be viewed primarily as a measure of tumor glycolysis rather than a direct surrogate of FDG uptake. In fact, in an analysis of a cohort of 20 breast tumor samples (100) we similarly observed that FDGScore, but not SUV_max_, was significantly negatively associated with the ssGSEA CD8 T cell signature (data not shown), suggesting that while FDGScore is associated with FDG uptake as measured by PET, they are not identical.

Tumor glycolysis is a critical component of tumor growth. In addition to fueling cell proliferation, it can directly regulate the mTOR pathway (82–84), regulate translation of immune-related mRNAs (79), and affect histone modification (85, 86). Additionally, increased tumor glycolysis and lactate production is known to directly inhibit effector T-cell function while promoting regulatory T-cell function (45). Our study has important limitations, such as the observational character of our analyses, the lack of validation of our findings at the protein level in larger cohorts, and the weak association of FDGScore and prognosis across multiple tumor types. However, we aimed to determine whether the association between tumor glycolysis and immune exclusion described in pre-clinical models of disease was also true across a wide range of solid human malignancies.

Despite the limitations mentioned, taken together our study indeed shows that tumor glycolysis is associated with exclusion of CD8 T-cells across most solid tumor types. In combination with the published literature demonstrating the causal effect of tumor glycolysis on immune exclusion in selected mouse models of disease, our study raises the interesting possibility that inhibiting tumor glycolysis may lead to increased immune cell infiltration across multiple solid tumor types, and thus may serve to increase the efficacy of immune checkpoint blockade. The combination of glycolysis inhibition in tumor cells with immune checkpoint blockade has been recently shown to lead to dramatically improved efficacy of ICB in mouse models of breast cancer and melanoma (45). In addition, an inhibitor of the lactate transporter MCT1 (AZD3965) has been shown to increase immune-cell infiltration into solid tumors in pre-clinical models (101), and has also entered phase I clinical trials, showing safety and on-target effects as measured by changes in urinary lactate (102). Whether inhibiting glycolysis and/or lactate transport in combination with ICB in highly glycolytic tumors will increase the efficacy of ICB in patients remains to be determined.

## Data availability statement

The original contributions presented in the study are included in the article/**Supplementary Material**. Further inquiries can be directed to the corresponding author.

## Ethics statement

The studies involving human participants were reviewed and approved by MSKCC IRB Protocol #17-236A. Written informed consent for participation was not required for this study in accordance with the national legislation and the institutional requirements.

## Author contributions

IC and RGB conceived the study. IC, FP and ES reviewed the MSK cases. FP and IC performed IHC analysis. IC performed the bioinformatics analyses. NS, RK, RS, and AD provided advice and guidance on the bioinformatic and biostatistical analyses. IC, FP and RGB wrote the manuscript, which was reviewed by all co-authors. RGB, ES and EM supervised the study. All authors contributed to the article and approved the submitted version.

## Funding

The authors wish to acknowledge assistance from the NCI R01-CA215136 grant, the Breast Research Fund at MSKCC, and the Cancer Center Support Grant P30 CA008748. FP is partially funded by an NIH/NCI P50 CA247749 grant.

## Acknowledgments

IC specifically thanks Dr. Jorge Reis-Filho for his conceptual assistance with the biostatistical analyses and guidance during his graduate studies and thesis writing, which led to the formulation of this manuscript. The authors also wish to thank Drs. Marina Asher and Irina Linkov for their assistance with IHC staining of primary breast tumor tissue.

## Conflict of interest

The authors declare that the research was conducted in the absence of any commercial or financial relationships that could be construed as a potential conflict of interest.

## Publisher’s note

All claims expressed in this article are solely those of the authors and do not necessarily represent those of their affiliated organizations, or those of the publisher, the editors and the reviewers. Any product that may be evaluated in this article, or claim that may be made by its manufacturer, is not guaranteed or endorsed by the publisher.

## Supplementary material

Supplementary Figure S1 Co-expression patterns of glycolysis and immune related genes within individual tumor types. **(A, B) **RNA expression data was downloaded from TCGA and other datasets (see Methods), and the correlation between expression of selected glycolysis and immune genes was plotted (red = positive correlation; blue = negative correlation. X marks correlation coefficients with p>0.05). Correlation profiles of mRNA expression of selected glycolysis- and immune-related genes across multiple solid tumor types from the TCGA and independent cohorts are shown **(A)**. **(B) **The expression of specific glycolysis and immune genes was plotted for specific tumor types and the Pearson and Spearman correlation coefficients were calculated.

Supplementary Figure S2 Co-expression patterns of glycolysis and immune related genes within specified subtypes of breast cancer, lung cancer, and melanoma. **(A-D)** Correlation profiles of mRNA expression of selected glycolysis- and immune-related genes across specific tumor subtypes from the TCGA are shown: A) AR-Normal vs. AR-High BRCA; B) BRAF WT vs. V600E MUT SKCM; C) EGFR WT vs. L858R MUT LUAD and D) KRAS WT vs. G12 MUT LUAD.

Supplementary Figure S3 Abundance of glycolytic proteins is associated with decreased immune infiltration. **(A) **Correlation profiles of protein abundance of selected glycolysis- and immune-related genes in selected tumor types from CPTAC. **(B)** The LDHA protein abundance was plotted against the percentage of immune cells in H&E-stained tumor biopsies [as reported in Xu, Zhang, et al. (50)]. Data is presented in the entire cohort *(left)*, and in the EGFR WT *(middle)* and MUT *(right)* cohorts. **(C)** LDHA protein abundance was plotted in samples classified as having low, middle or high levels of CD8 T cell infiltration as measured by IHC staining and scoring [as reported in Xu, Zhang, et al. (50)]. Data is presented in the entire cohort *(left)*, and separated into the individual EGFR WT *(middle)* and MUT *(right)* cohorts.

Supplementary Figure S4 Expression of FDGScore across clinical characteristics of multiple cohorts. **(A) **FDGScore expression was plotted in relation to the (i) tumor stage; (ii) patient age at diagnosis; and (iii) gender in the Pan Cancer TCGA cohort. **(B) **FDGScore expression was plotted in relation to (i) tumor stage and (ii) patient age at diagnosis in the breast METABRIC cohort. **(C) **FDGScore expression was plotted in relation to (i) tumor stage; (ii) patient age at diagnosis and (iii) gender in the GSE65904 Melanoma cohort. The relationship between FDGScore and Stage was determined by calculating a linear regression to obtain the B coefficient. The relationship between FDGScore and Age was determined by calculating a Spearman rank correlation to obtain the correlation coefficient. The relationship between FDGScore and Gender was determined by performing a t-test between FDGScore expression in Male/Female.

Supplementary Figure S5 Expression of FDGScore is negatively correlated with T cell estimates within individual tumor types. **(A-C) **The correlation profiles of the FDG uptake signature (FDGScore) and the estimates of T cell subset abundance (as measured by ssGSEA) were calculated and plotted for the ER-negative METABRIC (A), GSE65904 (B) and GSE119267 (C) cohorts *(left)*. The expression of the FDGScore vs. CD8 *(middle)* and Tcm *(right)* T cell estimates is also shown with the calculated Pearson and Spearman coefficients.

Supplementary Figure S6 Expression of FDGScore is negatively correlated with T cell estimates within Luminal and ER-positive breast tumor types. **(A-B) **The correlation profiles of the FDG uptake signature (FDGScore) and the estimates of T cell subset abundance (as measured by ssGSEA) were calculated and plotted for the Luminal A and Luminal B subtypes of the TCGA BRCA cohort (A) and for the ER-positive subtype of the METABRIC cohort (B) *(left)*. The expression of the FDGScore vs. CD8 *(middle)* and Tcm *(right)* T cell estimates is also shown with the calculated Pearson and Spearman coefficients.

Supplementary Figure S7 HM_Glycolysis expression is inversely correlated with estimates of CD8^+^ and T_cm_T cells across most solid tumor types. **(A-D) **The correlation profiles of the HM_Glycolysis signature and the estimates of T cell subset abundance (as measured by ssGSEA) were calculated and plotted for the entire TCGA Pan Cancer cohort (A) and for individual tumor types within TCGA (B-D) *(left)*. The expression of the HM_Glycolysis vs. CD8 *(middle)* and Tcm *(right)* T cell estimates is also shown with the calculated Pearson and Spearman coefficients.

Supplementary Figure S8 Expression of HM_Glycolysis is negatively correlated with T cell estimates within individual tumor types. **(A-C) **The correlation profiles of the HM_Glycolysis signature and the estimates of T cell subset abundance (as measured by ssGSEA) were calculated and plotted for the ER-negative METABRIC (A), GSE65904 (B) and GSE119267 (C) cohorts *(left)*. The expression of the FDGScore vs. CD8 *(middle)* and Tcm *(right)* T cell estimates is also shown with the calculated Pearson and Spearman coefficients.

Supplementary Figure S9 Expression of HM_Glycolysis is negatively correlated with T cell estimates within the Luminal and ER-positive breast tumor types.**(A-C) **The correlation profiles of the HM_Glycolysis signature and the estimates of T cell subset abundance (as measured by ssGSEA) were calculated and plotted for the TCGA BRCA LumA/LumB (A) and METABRIC ER-positive (B) cohorts. The expression of the FDGScore vs. CD8 *(middle)* and Tcm *(right)* T cell estimates is also shown with the calculated Pearson and Spearman coefficients.

Supplementary Figure S10 Glucose and lactate abundance in a cohort of 23 human breast tumors. **(A) **Glucose and lactate metabolite levels were obtained from the Tang et. al. dataset and the correlation between glucose, lactate, FDGScore and multiple T cell estimates were plotted. (**(B) **FDGScore expression was plotted vs. the levels of glucose and lactate. **(C) **Multiple T cell estimates were plotted vs. lactate abundance levels in the Tang dataset.

Supplementary Figure S11 Overall survival by FDGScore and the CD8 T cell estimate in individual subtypes of breast cancer. **(A-D) **Kaplan-Meier survival analysis was performed for the Luminal A/B (A) and Basal/Her2 (B) subtypes of the TCGA BRCA cohort; and for the ER-positive (C) and ER-negative (D) subtypes of the METABRIC cohort. The disease-specific survival probability of patients was measured in the top tertile vs the bottom tertile of expression of either FDGScore *(left)* or the CD8 T cell estimate *(right)* for each cancer type, and the Hazard Ratio (HR) was calculated.

Supplementary Figure S12 Overall survival by HM_Glycolysis in solid tumors. **(A-D)** Kaplan-Meier survival analysis was performed for the entire TCGA cohort (A), as well as individually for the TCGA SKCM (B), TCGA LUAD (C) and METABRIC (D) cohorts. The disease-specific survival probability of patients was measured in the top tertile vs the bottom tertile of expression of HM_Glycolysis for each cancer type, and the Hazard Ratio (HR) was calculated.

Supplementary Figure S13 Expression of glycolytic and immune markers in relation to FDG uptake in primary breast tumors. **(A)** Volumetric Regions of Interest (ROIs) were drawn on FDG-PET scans for 18 pts with available scans, and the SUV Peak was calculated and plotted against the GLUT1 H Score *(left)*, the LDHA H Score *(middle)* and the Mean Glycolysis H Score *(right)* in the MSK cohort of 49 patients with treatment-naïve ER-negative primary breast cancer; see Methods. **(B)** SUV Peak was plotted against the percentage of stromal TILs *(left)*, stromal CD3+ lymphocytes *(middle)* and stromal CD8+ lymphocytes *(right)*. **(C) **Recurrence-free survival was calculated and Kaplan-Meier plots were plotted for patients in the highest tertile of the Mean Glycolysis H Score *(left)* or stromal CD8+ lymphocytes *(right)* and compared to patients in the bottom 2 tertiles.

## References

[1] Galluzzi L Chan TA Kroemer G Wolchok JD López-Soto. A. The hallmarks of successful anticancer immunotherapy. Sci Trans Med (2018) 10:eaat7807. doi: 10.1126/scitranslmed.aat7807 30232229

[2] Kraehenbuehl L Weng C-H Eghbali S Wolchok JD Merghoub. T. Enhancing immunotherapy in cancer by targeting emerging immunomodulatory pathways. Nat Rev Clin Oncol (2022) 19:37–50. doi: 10.1038/s41571-021-00552-7 34580473

[3] Larkin J Chiarion-Sileni V Gonzalez R Grob J-J Rutkowski P Lao CD. Five-year survival with combined nivolumab and ipilimumab in advanced melanoma. New Engl J Med (2019) 381:1535–46. doi: 10.1056/NEJMoa1910836 31562797

[4] Reck M Rodríguez-Abreu D Robinson AG Hui R Csőszi T Fülöp A. Pembrolizumab versus chemotherapy for PD-L1–positive non–Small-Cell lung cancer. New Engl J Med (2016) 375:1823–33. doi: 10.1056/NEJMoa1606774 27718847

[5] Garon EB Rizvi NA Hui R Leighl N Balmanoukian AS Eder JP. Pembrolizumab for the treatment of non–Small-Cell lung cancer. New Engl J Med (2015) 372:2018–28. doi: 10.1056/NEJMoa1501824 25891174

[6] Sezer A Kilickap S Gümüş M Bondarenko I Özgüroğlu M Gogishvili M. Cemiplimab monotherapy for first-line treatment of advanced non-small-cell lung cancer with PD-L1 of at least 50%: A multicentre, open-label, global, phase 3, randomised, controlled trial. Lancet (2021) 397:592–604. doi: 10.1016/S0140-6736(21)00228-2 33581821

[7] Le DT Durham JN Smith KN Wang H Bartlett BR Aulakh LK. Mismatch repair deficiency predicts response of solid tumors to PD-1 blockade. Science (2017) 357:409–13. doi: 10.1126/science.aan6733 PMC5576142 28596308

[8] Le DT Uram JN Wang H Bjarne R Kemberling H. PD-1 blockade in tumors with mismatch-repair deficiency. New Engl J Med (2015) 372:2509–20. doi: 10.1056/NEJMoa1500596 PMC4481136 26028255

[9] André Thierry Shiu K-K Kim TW Jensen BV Jensen LH. Pembrolizumab in Microsatellite-Instability–high advanced colorectal cancer. New Engl J Med (2020) 383:2207–18. doi: 10.1056/NEJMoa2017699 33264544

[10] Hellmann MD Paz-Ares L Caro RB Zurawski B Kim S-W Costa EC. Nivolumab plus ipilimumab in advanced non–Small-Cell lung cancer. New Engl J Med (2019) 381:2020–31. doi: 10.1056/NEJMoa1910231 31562796

[11] Motzer RJ Tannir NM McDermott DF Frontera OArén Melichar B Choueiri TK. Nivolumab plus ipilimumab versus sunitinib in advanced renal-cell carcinoma. New Engl J Med (2018) 378:1277–90. doi: 10.1056/NEJMoa1712126 PMC5972549 29562145

[12] Hellmann MD Ciuleanu T-E Pluzanski A Lee JS Otterson GA Audigier-Valette C. Nivolumab plus ipilimumab in lung cancer with a high tumor mutational burden. New Engl J Med (2018) 378:2093–104. doi: 10.1056/NEJMoa1801946 PMC7193684 29658845

[13] Paz-Ares L Ciuleanu T-E Cobo M Schenker M Zurawski B Menezes J. First-line nivolumab plus ipilimumab combined with two cycles of chemotherapy in patients with non-small-cell lung cancer (CheckMate 9LA): an international, randomised, open-label, phase 3 trial. Lancet Oncol (2021) 22:198–211. doi: 10.1016/S1470-2045(20)30641-0 33476593

[14] Cortes J Cescon DW Rugo HS Nowecki Z Im S-A Yusof MMd. Pembrolizumab plus chemotherapy versus placebo plus chemotherapy for previously untreated locally recurrent inoperable or metastatic triple-negative breast cancer (KEYNOTE-355): A randomised, placebo-controlled, double-blind, phase 3 clinical trial. Lancet (2020) 396:1817–28. doi: 10.1016/S0140-6736(20)32531-9 33278935

[15] Cascone T William WN Weissferdt A Leung CH Lin HY Pataer A. Neoadjuvant nivolumab or nivolumab plus ipilimumab in operable non-small cell lung cancer: the phase 2 randomized NEOSTAR trial. Nat Med (2021) 27:504–14. doi: 10.1038/s41591-020-01224-2 PMC8818318 33603241

[16] Provencio M Nadal E Insa A García-Campelo MaríaR Casal-Rubio Joaquín Dómine M. Neoadjuvant chemotherapy and nivolumab in resectable non-small-cell lung cancer (NADIM): An open-label, multicentre, single-arm, phase 2 trial. Lancet Oncol (2020) 21:1413–22. doi: 10.1016/S1470-2045(20)30453-8 32979984

[17] Borghaei H Langer CJ Gadgeel S Papadimitrakopoulou VA Patnaik A Powell SF. 24-month overall survival from KEYNOTE-021 cohort G: Pemetrexed and carboplatin with or without pembrolizumab as first-line therapy for advanced nonsquamous non–small cell lung cancer. J Thorac Oncol (2019) 14:124–29. doi: 10.1016/j.jtho.2018.08.004 30138764

[18] Socinski MA Jotte RM Cappuzzo F Orlandi F Stroyakovskiy D Nogami N. Atezolizumab for first-line treatment of metastatic nonsquamous NSCLC. New Engl J Med (2018) 378:2288–301. doi: 10.1056/NEJMoa1716948 29863955

[19] Horn L Mansfield AS Szczęsna A Havel L Krzakowski M Hochmair MJ. First-line atezolizumab plus chemotherapy in extensive-stage small-cell lung cancer. New Engl J Med (2018) 379:2220–29. doi: 10.1056/NEJMoa1809064 30280641

[20] Schmid P Adams S Rugo HS Schneeweiss A Barrios CH Iwata H. Atezolizumab and nab-paclitaxel in advanced triple-negative breast cancer. N Engl J Med (2018) 379:2108–21. doi: 10.1056/NEJMoa1809615 30345906

[21] Eggermont AMM Blank CU Mandalà M Long GV Atkinson VG Dalle Stéphane. Adjuvant pembrolizumab versus placebo in resected stage III melanoma (EORTC 1325-MG/KEYNOTE-054): distant metastasis-free survival results from a double-blind, randomised, controlled, phase 3 trial. Lancet Oncol (2021) 22:643–54. doi: 10.1016/S1470-2045(21)00065-6 33857412

[22] Rozeman EA Menzies AM Akkooi ACJv Adhikari C Bierman C Wiel BAvde. Identification of the optimal combination dosing schedule of neoadjuvant ipilimumab plus nivolumab in macroscopic stage III melanoma (OpACIN-neo): a multicentre, phase 2, randomised, controlled trial. Lancet Oncol (2019) 20:948–60. doi: 10.1016/S1470-2045(19)30151-2 31160251

[23] Ascierto PA Vecchio MD Mandalá M Gogas H Arance AM Dalle S. Adjuvant nivolumab versus ipilimumab in resected stage IIIB–c and stage IV melanoma (CheckMate 238): 4-year results from a multicentre, double-blind, randomised, controlled, phase 3 trial. Lancet Oncol (2020) 21:1465–77. doi: 10.1016/S1470-2045(20)30494-0 32961119

[24] Zimmer L Livingstone E Hassel JC Fluck M Eigentler T Loquai C. Adjuvant nivolumab plus ipilimumab or nivolumab monotherapy versus placebo in patients with resected stage IV melanoma with no evidence of disease (IMMUNED): A randomised, double-blind, placebo-controlled, phase 2 trial. Lancet (2020) 395:1558–68. doi: 10.1016/S0140-6736(20)30417-7 32416781

[25] Kelly RJ Ajani JA Kuzdzal J Zander T Cutsem EV Piessen G. Adjuvant nivolumab in resected esophageal or gastroesophageal junction cancer. New Engl J Med (2021) 384:1191–203. doi: 10.1056/NEJMoa2032125 33789008

[26] Bajorin DF Witjes JA Gschwend JürgenE Schenker M Valderrama BegoñaP Tomita Y. Adjuvant nivolumab versus placebo in muscle-invasive urothelial carcinoma. New Engl J Med (2021) 384:2102–14. doi: 10.1056/NEJMoa2034442 PMC8215888 34077643

[27] Forde PM Chaft JE Smith KN Anagnostou V Cottrell TR Hellmann MD. Neoadjuvant PD-1 blockade in resectable lung cancer. New Engl J Med (2018) 378:1976–86. doi: 10.1056/NEJMoa1716078 PMC6223617 29658848

[28] Ribas A Wolchok. JD. Cancer immunotherapy using checkpoint blockade. Science (2018) 359:1350–55. doi: 10.1126/science.aar4060 PMC7391259 29567705

[29] Marcus L Lemery SJ Keegan P Pazdur R. FDA approval summary: Pembrolizumab for the treatment of microsatellite instability-high solid tumors. Clin Cancer Res: Clincanres (2019) 4070:2018. doi: 10.1158/1078-0432.CCR-18-4070 30787022

[30] Leone RD Powell JD. Metabolism of immune cells in cancer. Nat Rev Cancer (2020) 20:516–31. doi: 10.1038/s41568-020-0273-y PMC8041116 32632251

[31] Warburg O. The metabolism of carcinoma cells. J Cancer Res (1925) 9:148–63. doi: 10.1158/jcr.1925.148

[32] Shim H Dolde C Lewis BC Wu C-S Dang G Jungmann RA. c-myc transactivation of LDH-a: Implications for tumor metabolism and growth. Proc Natl Acad Sci (1997) 94:6658–63. doi: 10.1073/pnas.94.13.6658 PMC21214 9192621

[33] Elstrom RL Bauer DE Buzzai M Karnauskas R Harris MH Plas DR. Akt stimulates aerobic glycolysis in cancer cells. Cancer Res (2004) 64:3892–99. doi: 10.1158/0008-5472.CAN-03-2904 15172999

[34] Hall A Meyle KD Lange MK Klima M Sanderhoff M Dahl C. Dysfunctional oxidative phosphorylation makes malignant melanoma cells addicted to glycolysis driven by the (V600E)BRAF oncogene. Oncotarget (2013) 4:584–99. doi: 10.18632/oncotarget.965 PMC3720606 23603840

[35] Matoba S Kang J-G Patino WD Wragg A Boehm M Gavrilova O. p53 regulates mitochondrial respiration. Science (2006) 312:1650–53. doi: 10.1126/science.1126863 16728594

[36] Walenta S Wetterling M Lehrke M Schwickert G Sundfor K Rofstad EK. High lactate levels predict likelihood of metastases, tumor recurrence, and restricted patient survival in human cervical cancers. Cancer Res (2000) 60(4):916–21. 10706105

[37] Wike-Hooley JL van den Berg AP Zee Jvd Reinhold. HS. Human tumour pH and its variation. Eur J Cancer Clin Oncol (1985) 21:785–91. doi: 10.1016/0277-5379(85)90216-0 4043168

[38] Chang CH Qiu J O'Sullivan D Buck MD Noguchi T Curtis JD. Metabolic competition in the tumor microenvironment is a driver of cancer progression. Cell (2015) 162:1229–41. doi: 10.1016/j.cell.2015.08.016 PMC4864363 26321679

[39] Cohen IJ Blasberg R. Impact of the tumor microenvironment on tumor-infiltrating lymphocytes: Focus on breast cancer. Breast Cancer (Auckl) (2017) 11:1178223417731565. doi: 10.1177/1178223417731565 28979132 PMC5617083

[40] Serganova I Cohen IJ Vemuri K Shindo M Maeda M Mane M. LDH-a regulates the tumor microenvironment *via* HIF-signaling and modulates the immune response. PloS One (2018) 13:e0203965. doi: 10.1371/journal.pone.0203965 30248111 PMC6153000

[41] Brand A Singer K Koehl GE Kolitzus M Schoenhammer G Thiel A. LDHA-associated lactic acid production blunts tumor immunosurveillance by T and NK cells. Cell Metab (2016) 24:657–71. doi: 10.1016/j.cmet.2016.08.011 27641098

[42] Ho PC Bihuniak JD Macintyre AN Staron M Liu X Amezquita R. Phosphoenolpyruvate is a metabolic checkpoint of anti-tumor T cell responses. Cell (2015) 162:1217–28. doi: 10.1016/j.cell.2015.08.012 PMC4567953 26321681

[43] Angelin A Gil-de-Gómez L Dahiya S Jiao J Guo L Levine MH. Foxp3 reprograms T cell metabolism to function in low-glucose, high-lactate environments. Cell Metab (2017) 25:1282–93.e7. doi: 10.1016/j.cmet.2016.12.018 28416194 PMC5462872

[44] Watson MJ Vignali PDA Mullett SJ Overacre-Delgoffe AE Peralta RM Grebinoski S. Metabolic support of tumour-infiltrating regulatory T cells by lactic acid. Nature (2021) 591:645–51. doi: 10.1038/s41586-020-03045-2 PMC7990682 33589820

[45] Zappasodi R Serganova I Cohen IJ Maeda M Shindo M Senbabaoglu Y. CTLA-4 blockade drives loss of treg stability in glycolysis-low tumours. Nature (2021) 591:652–58. doi: 10.1038/s41586-021-03326-4 PMC8057670 33588426

[46] Zappasodi R Wolchok JD Merghoub T. Strategies for predicting response to checkpoint inhibitors. Curr Hematol Malig Rep (2018) 13:383–95. doi: 10.1007/s11899-018-0471-9 PMC6719799 30159703

[47] Hoadley KA Yau C Hinoue T Wolf DM Lazar AJ Drill E. Cell-of-Origin patterns dominate the molecular classification of 10,000 tumors from 33 types of cancer. Cell (2018) 173:291–304.e6. doi: 10.1016/j.cell.2018.03.022 29625048 PMC5957518

[48] Curtis C Shah SP Chin S-F Turashvili G Rueda OM Dunning MJ. The genomic and transcriptomic architecture of 2,000 breast tumours reveals novel subgroups. Nature (2012) 486:346–52. doi: 10.1038/nature10983 PMC3440846 22522925

[49] Cerami E Gao J Dogrusoz U Gross BE Sumer SO Aksoy BülentA. The cBio cancer genomics portal: An open platform for exploring multidimensional cancer genomics data. Cancer Discovery (2012) 2:401–04. doi: 10.1158/2159-8290.CD-12-0095 PMC3956037 22588877

[50] Xu J-Y Zhang C Wang X Zhai L Ma Y Mao Y. Integrative proteomic characterization of human lung adenocarcinoma. Cell (2020) 182:245–61.e17. doi: 10.1016/j.cell.2020.05.043 32649877

[51] Tang X Lin C-C Spasojevic I Iversen ES Chi J-T Marks. JR. A joint analysis of metabolomics and genetics of breast cancer. Breast Cancer Res (2014) 16:415. doi: 10.1186/s13058-014-0415-9 25091696 PMC4187326

[52] Palaskas N Larson SM Schultz N Komisopoulou E Wong J Rohle D. 18F-fluorodeoxy-glucose positron emission tomography marks MYC-overexpressing human basal-like breast cancers. Cancer Res (2011) 71:5164–74. doi: 10.1158/0008-5472.CAN-10-4633 PMC3148325 21646475

[53] Senbabaoglu Y Gejman RS Winer AG Liu M Van Allen EM de Velasco G. Tumor immune microenvironment characterization in clear cell renal cell carcinoma identifies prognostic and immunotherapeutically relevant messenger RNA signatures. Genome Biol (2016) 17:231. doi: 10.1186/s13059-016-1092-z 27855702 PMC5114739

[54] Liberzon A Birger C Thorvaldsdottir H Ghandi M Mesirov JP Tamayo P. The molecular signatures database (MSigDB) hallmark gene set collection. Cell Syst (2015) 1:417–25. doi: 10.1016/j.cels.2015.12.004 PMC4707969 26771021

[55] Liu J Lichtenberg T Hoadley KA Poisson LM Lazar AJ Cherniack AD. An integrated TCGA pan-cancer clinical data resource to drive high-quality survival outcome analytics. Cell (2018) 173:400–16.e11. doi: 10.1016/j.cell.2018.02.052 29625055 PMC6066282

[56] Tumours B. WHO classification of tumours. Lyon France: Int Agency Res Canc (2019).

[57] Elston CW Ellis IO. Pathological prognostic factors in breast cancer. i. the value of histological grade in breast cancer: Experience from a large study with long-term follow-up. Histopathology (1991) 19:403–10. doi: 10.1111/j.1365-2559.1991.tb00229.x 1757079

[58] Salgado R Denkert C Demaria S Sirtaine N Klauschen F Pruneri G. The evaluation of tumor-infiltrating lymphocytes (TILs) in breast cancer: recommendations by an international TILs working group 2014. Ann Oncol (2015) 26:259–71. doi: 10.1093/annonc/mdu450 PMC6267863 25214542

[59] Pareja F Toss MS Geyer FC da Silva EM Vahdatinia M Sebastiao APM. Immunohistochemical assessment of HRAS Q61R mutations in breast adenomyoepitheliomas. Histopathology (2020) 76:865–74. doi: 10.1111/his.14057 PMC7225035 31887226

[60] Pareja F da Silva EM Frosina D Geyer FC Lozada JR Basili T. Immunohistochemical analysis of IDH2 R172 hotspot mutations in breast papillary neoplasms: Applications in the diagnosis of tall cell carcinoma with reverse polarity. Modern Pathol (2020) 33:1056–64. doi: 10.1038/s41379-019-0442-2 PMC7286791 31896809

[61] Winer EP Lipatov O Im S-A Goncalves A Muñoz-Couselo E Lee KS. Pembrolizumab versus investigator-choice chemotherapy for metastatic triple-negative breast cancer (KEYNOTE-119): A randomised, open-label, phase 3 trial. Lancet Oncol (2021) 22:499–511. doi: 10.1016/S1470-2045(20)30754-3 33676601

[62] Kojima T Shah MA Muro K Francois E Adenis A Hsu C-H. Randomized phase III KEYNOTE-181 study of pembrolizumab versus chemotherapy in advanced esophageal cancer. J Clin Oncol (2020) 38:4138–48. doi: 10.1200/JCO.20.01888 33026938

[63] Shitara K Cutsem EV Bang Y-J Fuchs C Wyrwicz L Lee K-W. Efficacy and safety of pembrolizumab or pembrolizumab plus chemotherapy vs chemotherapy alone for patients with first-line, advanced gastric cancer: The KEYNOTE-062 phase 3 randomized clinical trial. JAMA Oncol (2020) 6:1571–80. doi: 10.1001/jamaoncol.2020.3370 PMC7489405 32880601

[64] Marabelle Aurélien Fakih M Lopez J Shah M Shapira-Frommer R Nakagawa K. Association of tumour mutational burden with outcomes in patients with advanced solid tumours treated with pembrolizumab: prospective biomarker analysis of the multicohort, open-label, phase 2 KEYNOTE-158 study. Lancet Oncol (2020) 21:1353–65. doi: 10.1016/S1470-2045(20)30445-9 32919526

[65] Feng J Li J Wu L Yu Q Ji J Wu J. Emerging roles and the regulation of aerobic glycolysis in hepatocellular carcinoma. J Exp Clin Cancer Res (2020) 39:126. doi: 10.1186/s13046-020-01629-4 32631382 PMC7336654

[66] Wu Z Wu J Zhao Q Fu S Jin J. Emerging roles of aerobic glycolysis in breast cancer. Clin Transl Oncol (2020) 22:631–46. doi: 10.1007/s12094-019-02187-8 31359335

[67] Cirenajwis H Ekedahl H Lauss M Harbst K Carneiro A Enoksson J. Molecular stratification of metastatic melanoma using gene expression profiling: Prediction of survival outcome and benefit from molecular targeted therapy. Oncotarget (2015) 6:12297–309. doi: 10.18632/oncotarget.3655 PMC4494939 25909218

[68] Subat S Inamura K Ninomiya H Nagano H Okumura S Ishikawa. Y. Unique MicroRNA and mRNA interactions in EGFR-mutated lung adenocarcinoma. J Clin Med (2018) 7:419. doi: 10.3390/jcm7110419 30404194 PMC6262391

[69] Vasaikar S Huang C Wang X Petyuk VA Savage SR Wen Bo. Proteogenomic analysis of human colon cancer reveals new therapeutic opportunities. Cell (2019) 177:1035–49.e19. doi: 10.1016/j.cell.2019.03.030 31031003 PMC6768830

[70] Gillette MA Satpathy S Cao S Dhanasekaran SM Vasaikar SV Krug K. Proteogenomic characterization reveals therapeutic vulnerabilities in lung adenocarcinoma. Cell (2020) 182:200–25.e35. doi: 10.1016/j.cell.2020.06.013 32649874 PMC7373300

[71] Dou Y Kawaler EA Cui Zhou D Gritsenko MA Huang C Blumenberg L. Proteogenomic characterization of endometrial carcinoma. Cell (2020) 180:729–48.e26. doi: 10.1016/j.cell.2020.01.026 32059776 PMC7233456

[72] Wang LB Karpova A Gritsenko MA Kyle JE Cao S Li Y. Proteogenomic and metabolomic characterization of human glioblastoma. Cancer Cell (2021) 39:509–28.e20. doi: 10.1016/j.ccell.2021.01.006 33577785 PMC8044053

[73] Satpathy S Krug K Jean Beltran PM Savage SR Petralia F Kumar-Sinha C. A proteogenomic portrait of lung squamous cell carcinoma. Cell (2021) 184:4348–71.e40. doi: 10.1016/j.cell.2021.07.016 34358469 PMC8475722

[74] Cao L Huang C Cui Zhou D Hu Y Lih TM Savage SR. Proteogenomic characterization of pancreatic ductal adenocarcinoma. Cell (2021) 184:5031–52.e26. doi: 10.1016/j.cell.2021.08.023 34534465 PMC8654574

[75] Krug K Jaehnig EJ Satpathy S Blumenberg L Karpova A Anurag M. Proteogenomic landscape of breast cancer tumorigenesis and targeted therapy. Cell (2020) 183:1436–56.e31. doi: 10.1016/j.cell.2020.10.036 33212010 PMC8077737

[76] Zhang H Liu T Zhang Z Payne SH Zhang B McDermott JE. Integrated proteogenomic characterization of human high-grade serous ovarian cancer. Cell (2016) 166:755–65. doi: 10.1016/j.cell.2016.05.069 PMC4967013 27372738

[77] Hakimi A Attalla K DiNatale RG Ostrovnaya I Flynn J. A pan-cancer analysis of PBAF complex mutations and their association with immunotherapy response. Nat Commun (2020) 11:4168. doi: 10.1038/s41467-020-17965-0 32820162 PMC7441387

[78] Fischer K Hoffmann P Voelkl S Meidenbauer N Ammer J Edinger M. Inhibitory effect of tumor cell–derived lactic acid on human T cells. (2007) 109(9):3812–9. doi: 10.1182/blood-2006-07-035972 17255361

[79] Chang C-H Curtis JD Maggi LB Faubert B Villarino AV O’Sullivan D. Posttranscriptional control of T cell effector function by aerobic glycolysis. Cell (2013) 153:1239–51. doi: 10.1016/j.cell.2013.05.016 PMC3804311 23746840

[80] Warburg O Wind F Negelein E. THE METABOLISM OF TUMORS IN THE BODY. J Gen Physiol (1927) 8:519–30. doi: 10.1085/jgp.8.6.519 PMC2140820 19872213

[81] Ward PS Thompson CB. Metabolic reprogramming: a cancer hallmark even warburg did not anticipate. Cancer Cell (2012) 21:297–308. doi: 10.1016/j.ccr.2012.02.014 22439925 PMC3311998

[82] Orozco JM Krawczyk PA Scaria SM Cangelosi AL Chan SH Kunchok T. Dihydroxyacetone phosphate signals glucose availability to mTORC1. Nat Metab (2020) 2:893–901. doi: 10.1038/s42255-020-0250-5 32719541 PMC7995735

[83] Xu Ke Yin Na Peng M Stamatiades EG Shyu A Li P. Glycolysis fuels phosphoinositide 3-kinase signaling to bolster T cell immunity. Science (2021) 371:405–10. doi: 10.1126/science.abb2683 PMC8380312 33479154

[84] Haas R Cucchi D Smith J Pucino V Macdougall CE Mauro. C. Intermediates of metabolism: From bystanders to signalling molecules. Trends Biochem Sci (2016) 41:460–71. doi: 10.1016/j.tibs.2016.02.003 26935843

[85] Peng M Yin Na Chhangawala S Xu Ke Leslie CS Li. MO. Aerobic glycolysis promotes T helper 1 cell differentiation through an epigenetic mechanism. Science (2016) 354:481–84. doi: 10.1126/science.aaf6284 PMC5539971 27708054

[86] Zhang Di Tang Z Huang He Zhou G Cui C Weng Y. Metabolic regulation of gene expression by histone lactylation. Nature (2019) 574:575–80. doi: 10.1038/s41586-019-1678-1 PMC6818755 31645732

[87] Yao J Li R Liu X Zhou X Li J Liu T. Prognostic implication of glycolysis related gene signature in non-small cell lung cancer. J Cancer (2021) 12:885–98. doi: 10.7150/jca.50274 PMC7778529 33403045

[88] Zhang L Zhang Z Yu Z. Identification of a novel glycolysis-related gene signature for predicting metastasis and survival in patients with lung adenocarcinoma. J Transl Med (2019) 17:423. doi: 10.1186/s12967-019-02173-2 31847905 PMC6916245

[89] Tang J Luo Y Wu G. A glycolysis-related gene expression signature in predicting recurrence of breast cancer. Aging (Albany NY) (2020) 12:24983–94. doi: 10.18632/aging.103806 PMC7803557 33201835

[90] Yu S Hu C Cai L Du X Lin F Yu Q. Seven-gene signature based on glycolysis is closely related to the prognosis and tumor immune infiltration of patients with gastric cancer. Front Oncol (2020) 10(1778). doi: 10.3389/fonc.2020.01778 PMC7531434 33072557

[91] Liu J Li S Feng G Meng H Nie S Sun R. Nine glycolysis-related gene signature predicting the survival of patients with endometrial adenocarcinoma. Cancer Cell Int (2020) 20:183. doi: 10.1186/s12935-020-01264-1 32489319 PMC7247270

[92] Jiang F Wu C Wang M Wei K Wang J. Identification of novel cell glycolysis related gene signature predicting survival in patients with breast cancer. Sci Rep (2021) 11(1):3986. doi: 10.1038/s41598-021-83628-9 33597614 PMC7889867

[93] Xu F Guan Y Xue L Huang S Gao K Yang Z. The effect of a novel glycolysis-related gene signature on progression, prognosis and immune microenvironment of renal cell carcinoma. BMC Cancer (2020) 20(1):1207. doi: 10.1186/s12885-020-07702-7 33287763 PMC7720455

[94] Wei J Huang K Chen Z Hu M Bai Y Lin S. Characterization of glycolysis-associated molecules in the tumor microenvironment revealed by pan-cancer tissues and lung cancer single cell data. Cancers (Basel) (2020) 12(7):1788. doi: 10.3390/cancers12071788 32635458 PMC7408567

[95] Wu Z Wen Z Li Z Yu M Ye G. Identification and prognostic value of a glycolysis-related gene signature in patients with bladder cancer. Med (Baltimore) (2021) 100(3):e23836. doi: 10.1097/MD.0000000000023836 PMC7837905 33545950

[96] Sturm G Finotello F Petitprez F Zhang JD Baumbach J Fridman WH. Comprehensive evaluation of transcriptome-based cell-type quantification methods for immuno-oncology. Bioinformatics (2019) 35(14):i436–i445. doi: 10.1093/bioinformatics/btz363 PMC6612828 31510660

[97] Harbison RA Pandey R Considine M Leone RD Murray-Stewart T Erbe R. Interrogation of T cell-enriched tumors reveals prognostic and immunotherapeutic implications of polyamine metabolism. Cancer Res Commun (2022) 2:639–52. doi: 10.1158/2767-9764.CRC-22-0061 PMC9432485 36052016

[98] Murakami W Tozaki M Sasaki M Hida AI Ohi Y Kubota K. Correlation between (18)F-FDG uptake on PET/MRI and the level of tumor-infiltrating lymphocytes (TILs) in triple-negative and HER2-positive breast cancer. Eur J Radiol (2020) 123:108773. doi: 10.1016/j.ejrad.2019.108773 31918248

[99] An YS Kim SH Roh TH Park SH Kim TG Kim JH. Correlation between (18)F-FDG uptake and immune cell infiltration in metastatic brain lesions. Front Oncol (2021) 11:618705. doi: 10.3389/fonc.2021.618705 34249674 PMC8266210

[100] Osborne JR Port E Gonen M Doane A Yeung H Gerald W. 18F-FDG PET of locally invasive breast cancer and association of estrogen receptor status with standardized uptake value: Microarray and immunohistochemical analysis. J Nucl Med (2010) 51:543–50. doi: 10.2967/jnumed.108.060459 PMC4141648 20237034

[101] Beloueche-Babari M Galobart TC Delgado-Goni T Wantuch S Parkes HG Tandy D. Monocarboxylate transporter 1 blockade with AZD3965 inhibits lipid biosynthesis and increases tumour immune cell infiltration. Br J Cancer (2020) 122:895–903. doi: 10.1038/s41416-019-0717-x 31937921 PMC7078321

[102] Halford SER Jones P Wedge S Hirschberg S Katugampola S Veal G. A first-in-human first-in-class (FIC) trial of the monocarboxylate transporter 1 (MCT1) inhibitor AZD3965 in patients with advanced solid tumours. J Clin Oncol (2017) 35:2516–16. doi: 10.1200/JCO.2017.35.15_suppl.2516

