# Supplementary figures and images for: Increased tumor glycolysis is associated with decreased immune infiltration across human solid tumors
Source: Front Immunol. 2022 Nov 24;13:880959. doi: 10.3389/fimmu.2022.880959 (PMC9731115; doi:10.3389/fimmu.2022.880959)

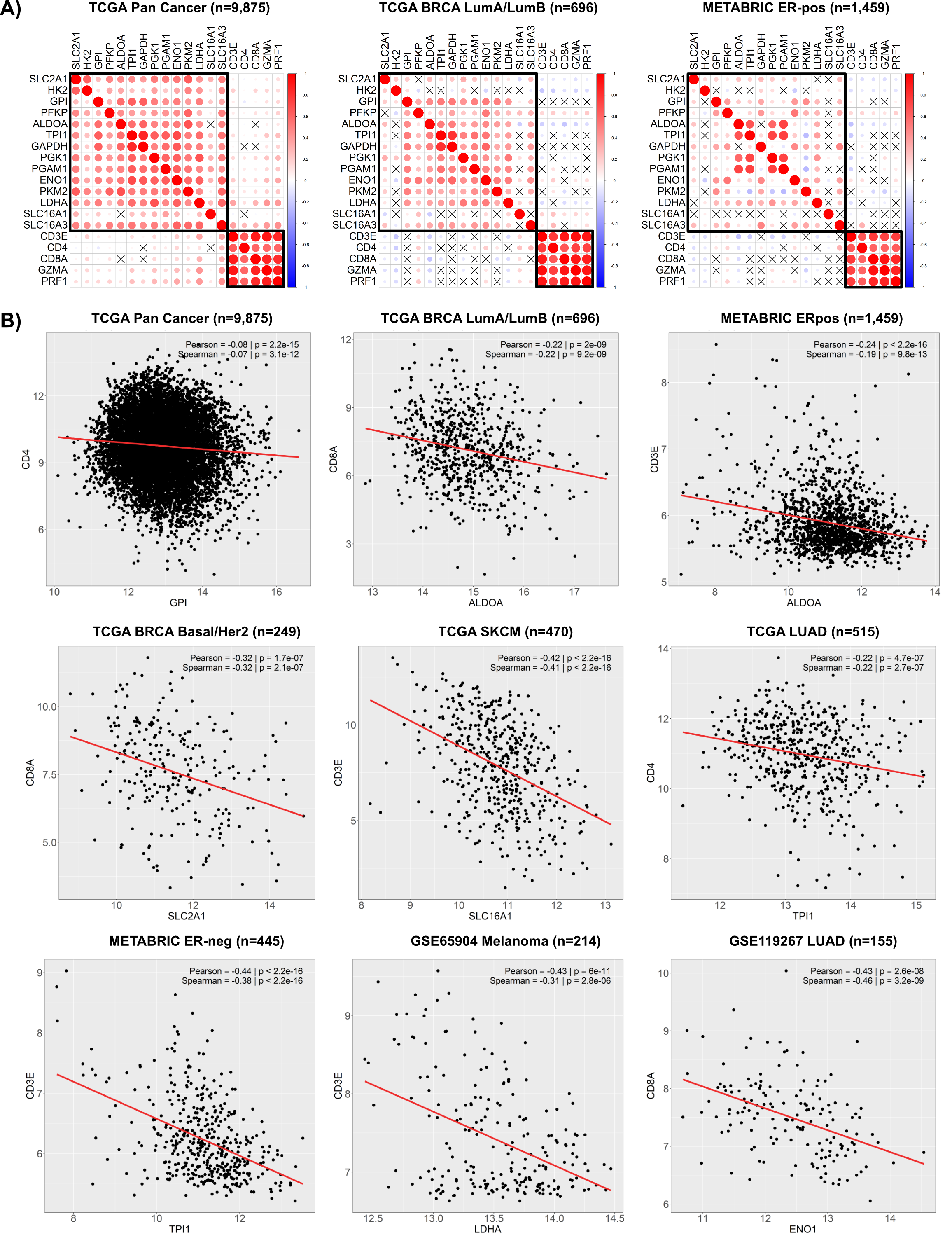

Supplement: Supplementary Figure S1 — Co-expression patterns of glycolysis and immune related genes within individual tumor types. (A, B) RNA expression data was downloaded from TCGA and other datasets (see Methods), and the correlation between expression of selected glycolysis and immune genes was plotted (red = positive correlation; blue = negative correlation. X marks correlation coefficients with p>0.05). Correlation profiles of mRNA expression of selected glycolysis- and immune-related genes across multiple solid tumor types from the TCGA and independent cohorts are shown (A). (B) The expression of specific glycolysis and immune genes was plotted for specific tumor types and the Pearson and Spearman correlation coefficients were calculated. [file DataSheet_1.zip › Supplementary Figures/Supp Figure S1.tiff]

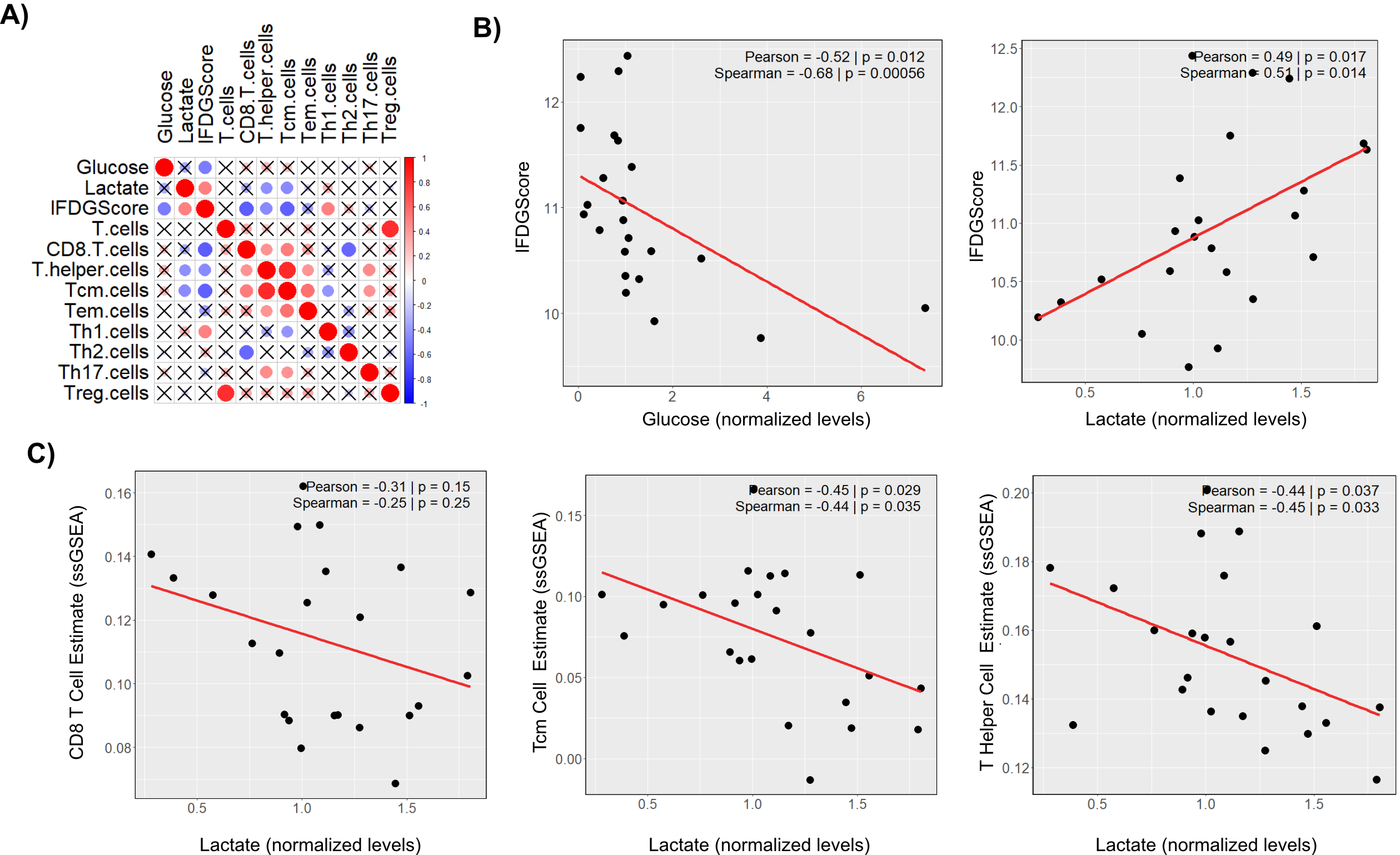

Supplement: Supplementary Figure S1 — Co-expression patterns of glycolysis and immune related genes within individual tumor types. (A, B) RNA expression data was downloaded from TCGA and other datasets (see Methods), and the correlation between expression of selected glycolysis and immune genes was plotted (red = positive correlation; blue = negative correlation. X marks correlation coefficients with p>0.05). Correlation profiles of mRNA expression of selected glycolysis- and immune-related genes across multiple solid tumor types from the TCGA and independent cohorts are shown (A). (B) The expression of specific glycolysis and immune genes was plotted for specific tumor types and the Pearson and Spearman correlation coefficients were calculated. [file DataSheet_1.zip › Supplementary Figures/Supp Figure S10.tiff]

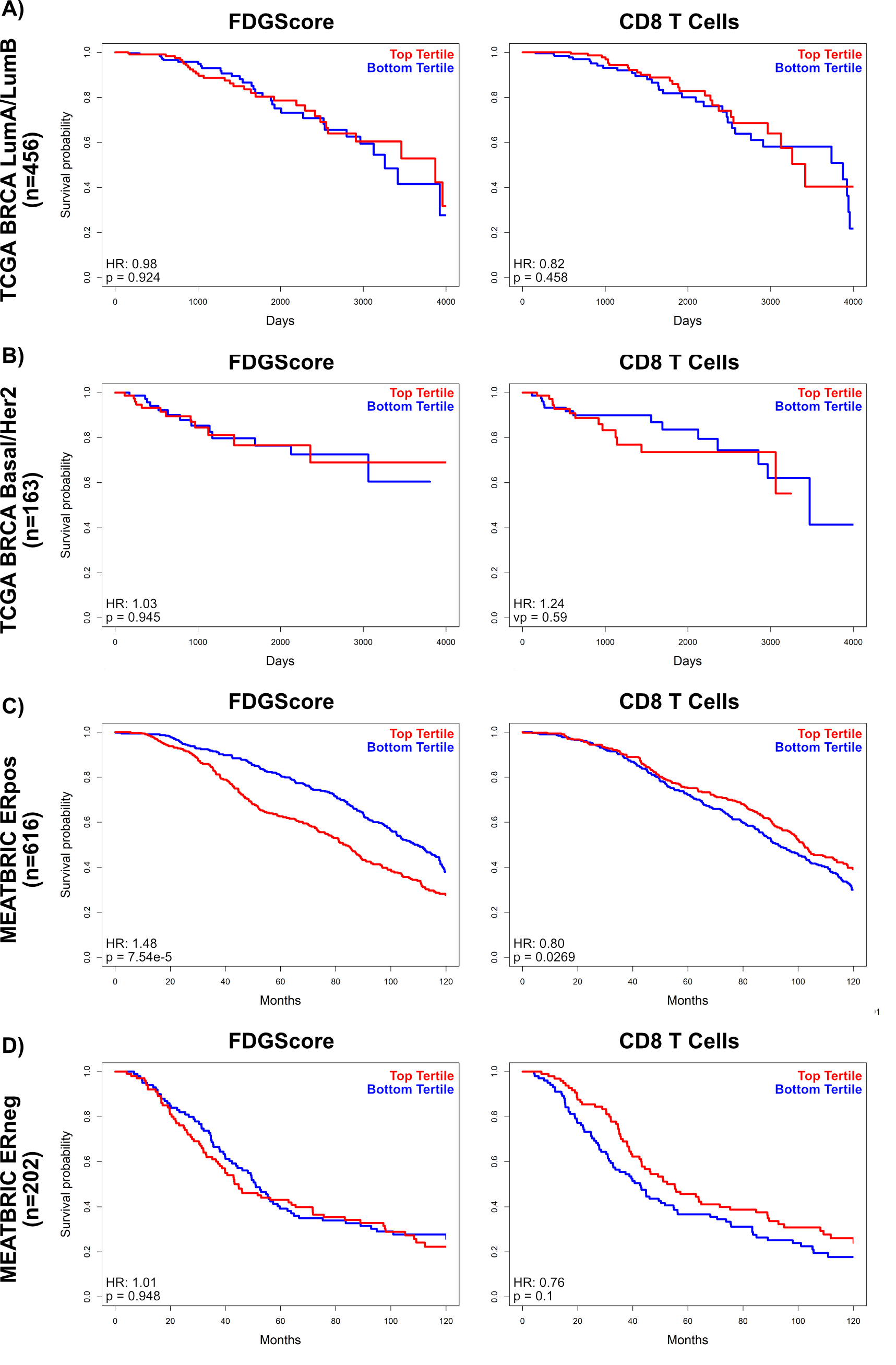

Supplement: Supplementary Figure S1 — Co-expression patterns of glycolysis and immune related genes within individual tumor types. (A, B) RNA expression data was downloaded from TCGA and other datasets (see Methods), and the correlation between expression of selected glycolysis and immune genes was plotted (red = positive correlation; blue = negative correlation. X marks correlation coefficients with p>0.05). Correlation profiles of mRNA expression of selected glycolysis- and immune-related genes across multiple solid tumor types from the TCGA and independent cohorts are shown (A). (B) The expression of specific glycolysis and immune genes was plotted for specific tumor types and the Pearson and Spearman correlation coefficients were calculated. [file DataSheet_1.zip › Supplementary Figures/Supp Figure S11.tiff]

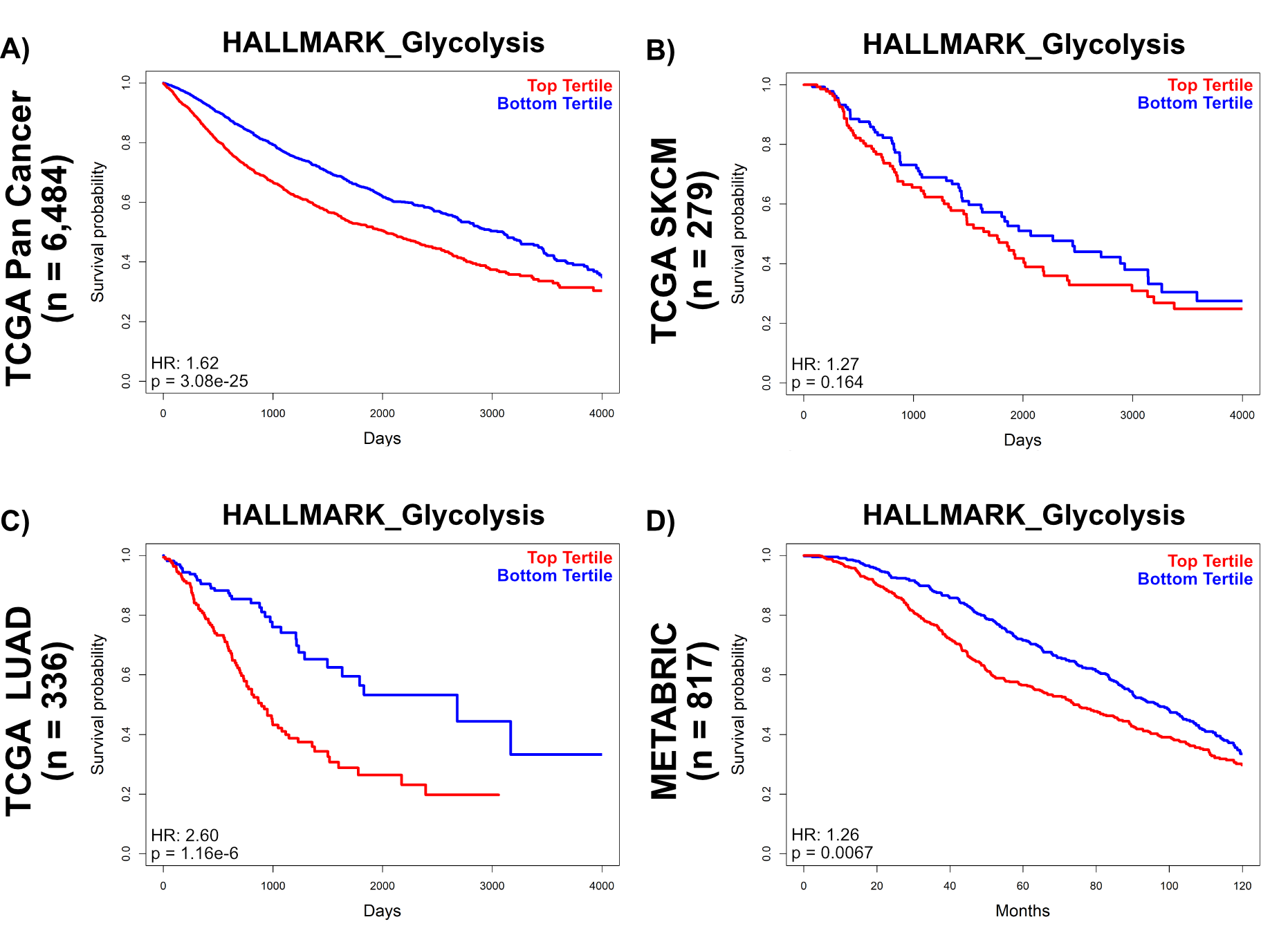

Supplement: Supplementary Figure S1 — Co-expression patterns of glycolysis and immune related genes within individual tumor types. (A, B) RNA expression data was downloaded from TCGA and other datasets (see Methods), and the correlation between expression of selected glycolysis and immune genes was plotted (red = positive correlation; blue = negative correlation. X marks correlation coefficients with p>0.05). Correlation profiles of mRNA expression of selected glycolysis- and immune-related genes across multiple solid tumor types from the TCGA and independent cohorts are shown (A). (B) The expression of specific glycolysis and immune genes was plotted for specific tumor types and the Pearson and Spearman correlation coefficients were calculated. [file DataSheet_1.zip › Supplementary Figures/Supp Figure S12.tiff]

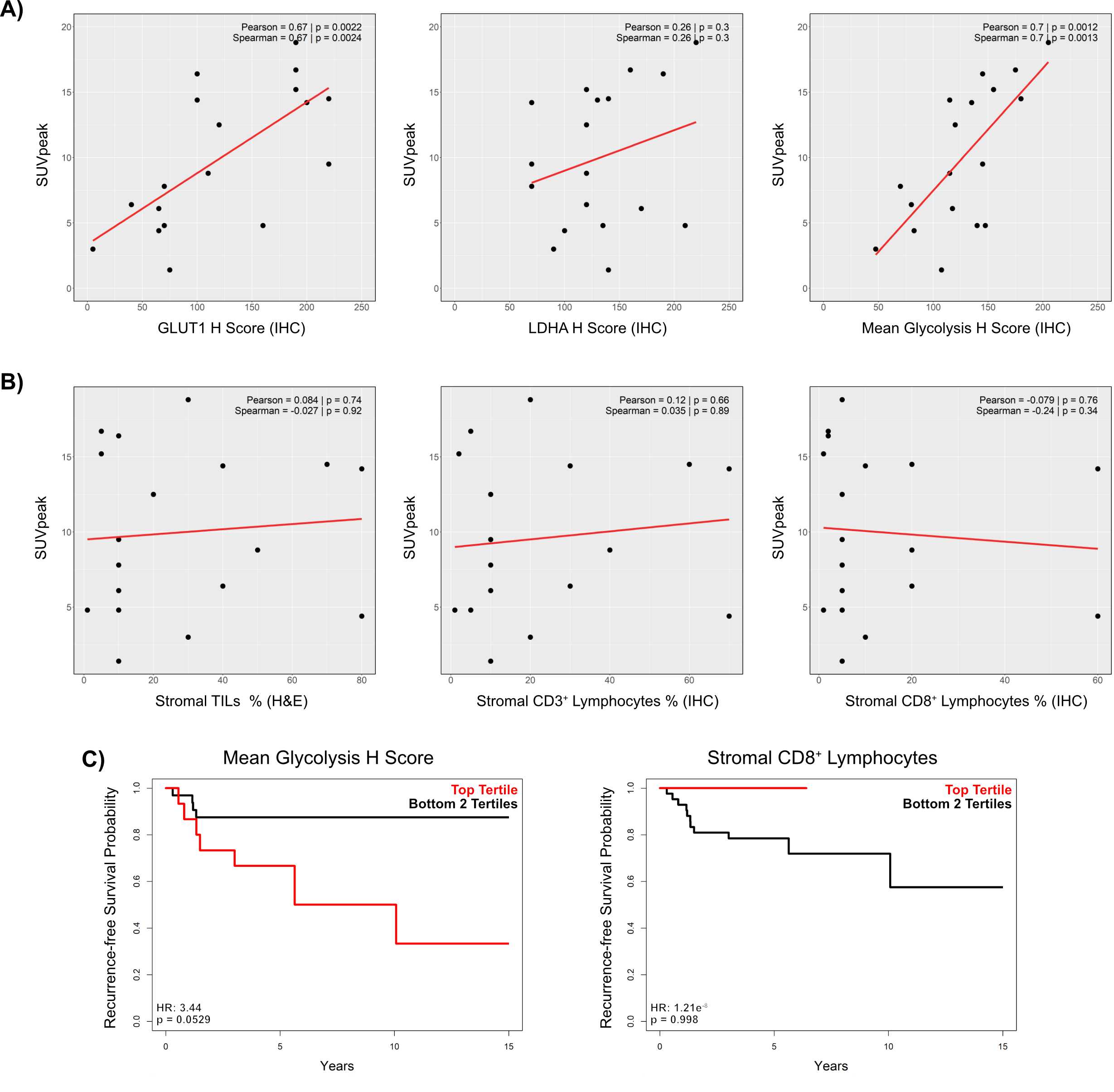

Supplement: Supplementary Figure S1 — Co-expression patterns of glycolysis and immune related genes within individual tumor types. (A, B) RNA expression data was downloaded from TCGA and other datasets (see Methods), and the correlation between expression of selected glycolysis and immune genes was plotted (red = positive correlation; blue = negative correlation. X marks correlation coefficients with p>0.05). Correlation profiles of mRNA expression of selected glycolysis- and immune-related genes across multiple solid tumor types from the TCGA and independent cohorts are shown (A). (B) The expression of specific glycolysis and immune genes was plotted for specific tumor types and the Pearson and Spearman correlation coefficients were calculated. [file DataSheet_1.zip › Supplementary Figures/Supp Figure S13.tiff]

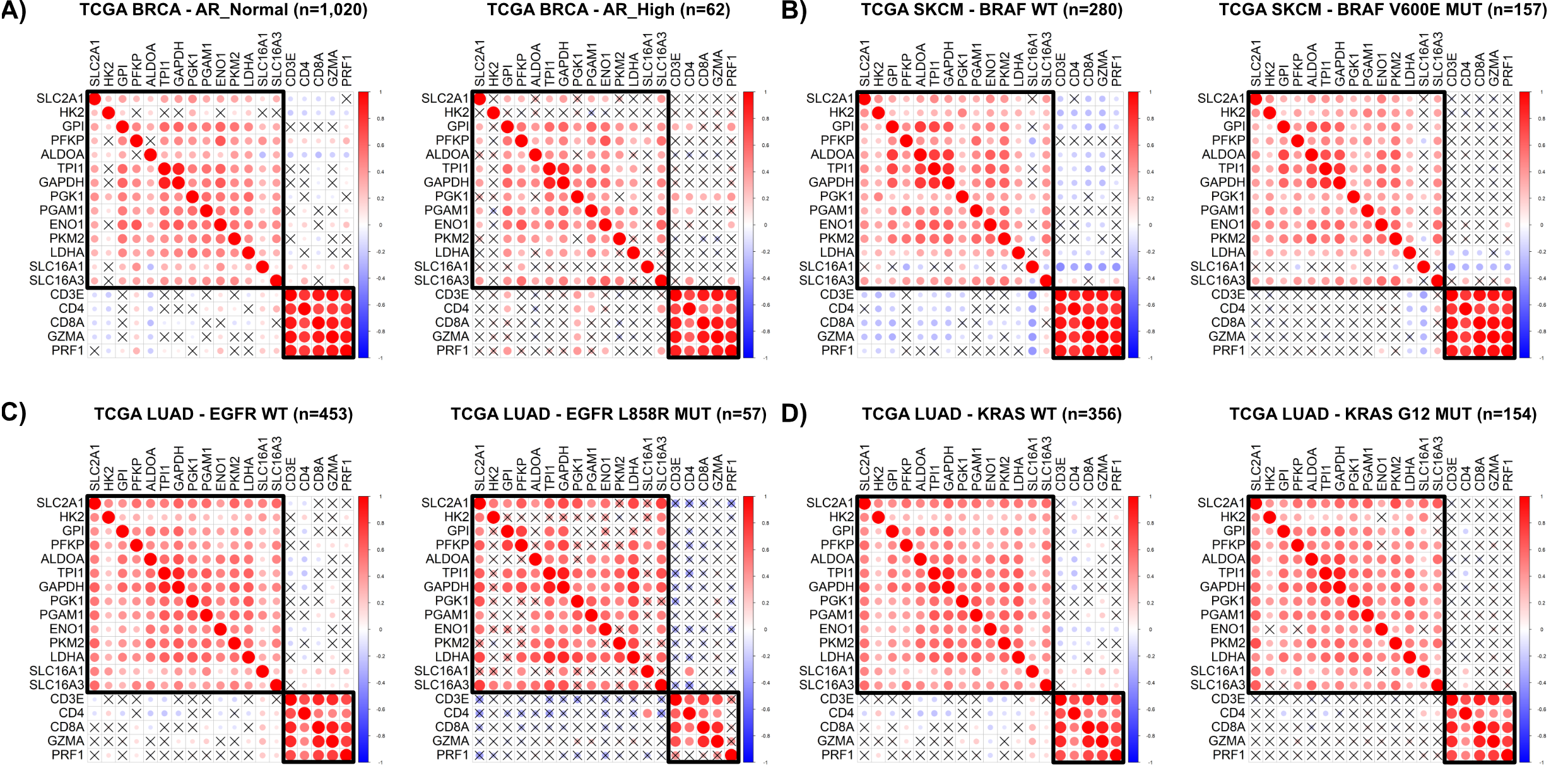

Supplement: Supplementary Figure S1 — Co-expression patterns of glycolysis and immune related genes within individual tumor types. (A, B) RNA expression data was downloaded from TCGA and other datasets (see Methods), and the correlation between expression of selected glycolysis and immune genes was plotted (red = positive correlation; blue = negative correlation. X marks correlation coefficients with p>0.05). Correlation profiles of mRNA expression of selected glycolysis- and immune-related genes across multiple solid tumor types from the TCGA and independent cohorts are shown (A). (B) The expression of specific glycolysis and immune genes was plotted for specific tumor types and the Pearson and Spearman correlation coefficients were calculated. [file DataSheet_1.zip › Supplementary Figures/Supp Figure S2.tiff]

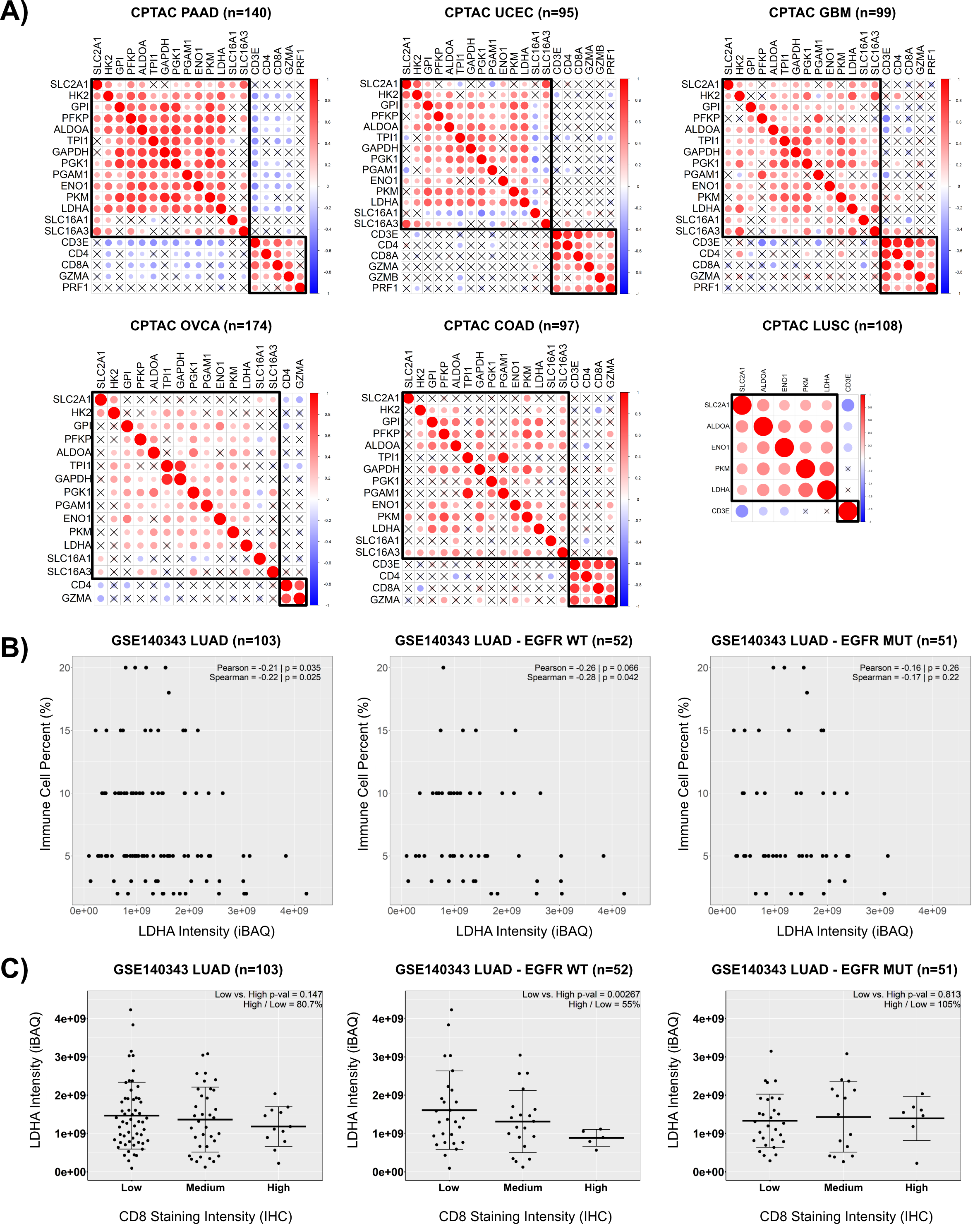

Supplement: Supplementary Figure S1 — Co-expression patterns of glycolysis and immune related genes within individual tumor types. (A, B) RNA expression data was downloaded from TCGA and other datasets (see Methods), and the correlation between expression of selected glycolysis and immune genes was plotted (red = positive correlation; blue = negative correlation. X marks correlation coefficients with p>0.05). Correlation profiles of mRNA expression of selected glycolysis- and immune-related genes across multiple solid tumor types from the TCGA and independent cohorts are shown (A). (B) The expression of specific glycolysis and immune genes was plotted for specific tumor types and the Pearson and Spearman correlation coefficients were calculated. [file DataSheet_1.zip › Supplementary Figures/Supp Figure S3.tiff]

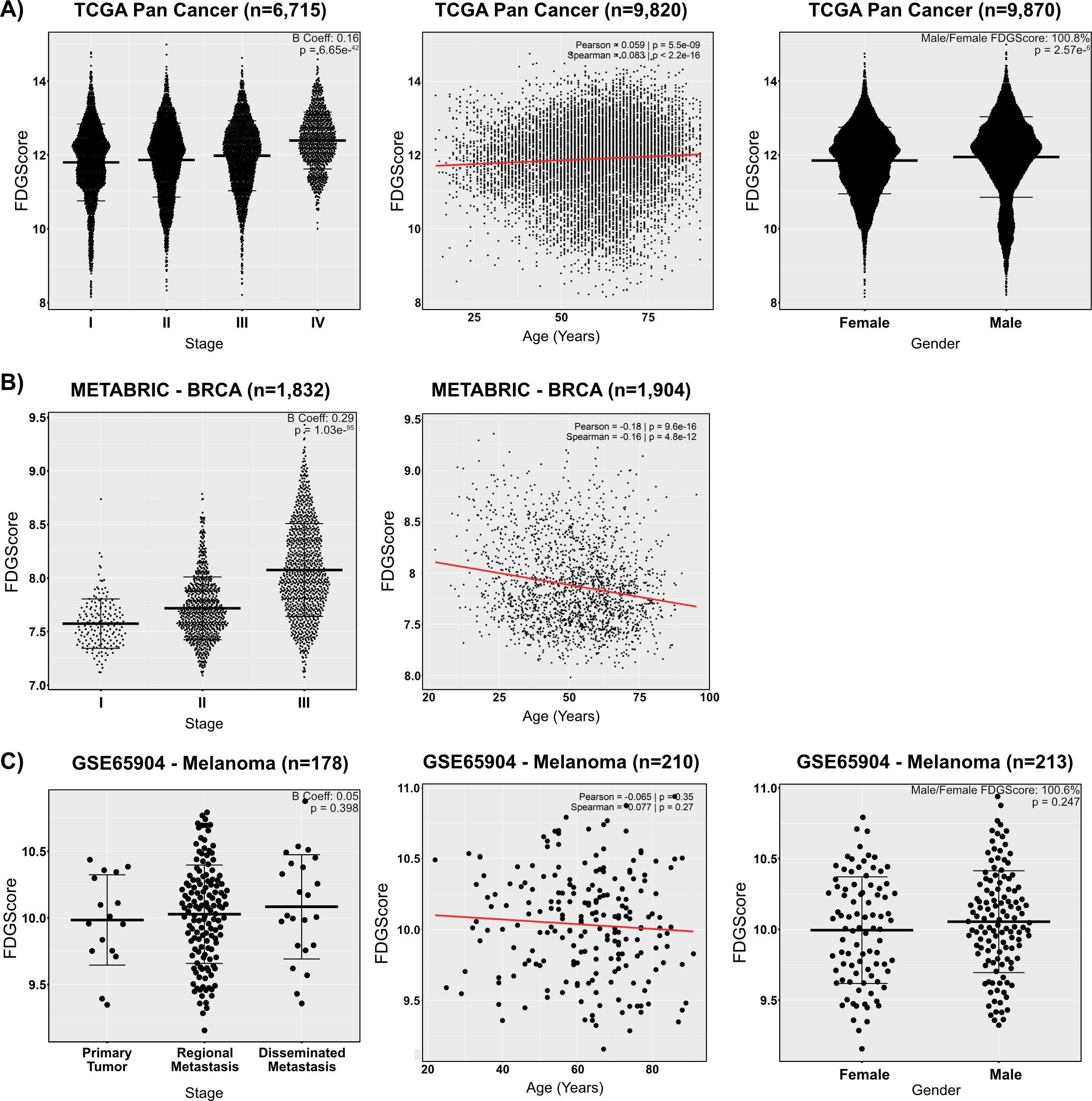

Supplement: Supplementary Figure S1 — Co-expression patterns of glycolysis and immune related genes within individual tumor types. (A, B) RNA expression data was downloaded from TCGA and other datasets (see Methods), and the correlation between expression of selected glycolysis and immune genes was plotted (red = positive correlation; blue = negative correlation. X marks correlation coefficients with p>0.05). Correlation profiles of mRNA expression of selected glycolysis- and immune-related genes across multiple solid tumor types from the TCGA and independent cohorts are shown (A). (B) The expression of specific glycolysis and immune genes was plotted for specific tumor types and the Pearson and Spearman correlation coefficients were calculated. [file DataSheet_1.zip › Supplementary Figures/Supp Figure S4.tiff]

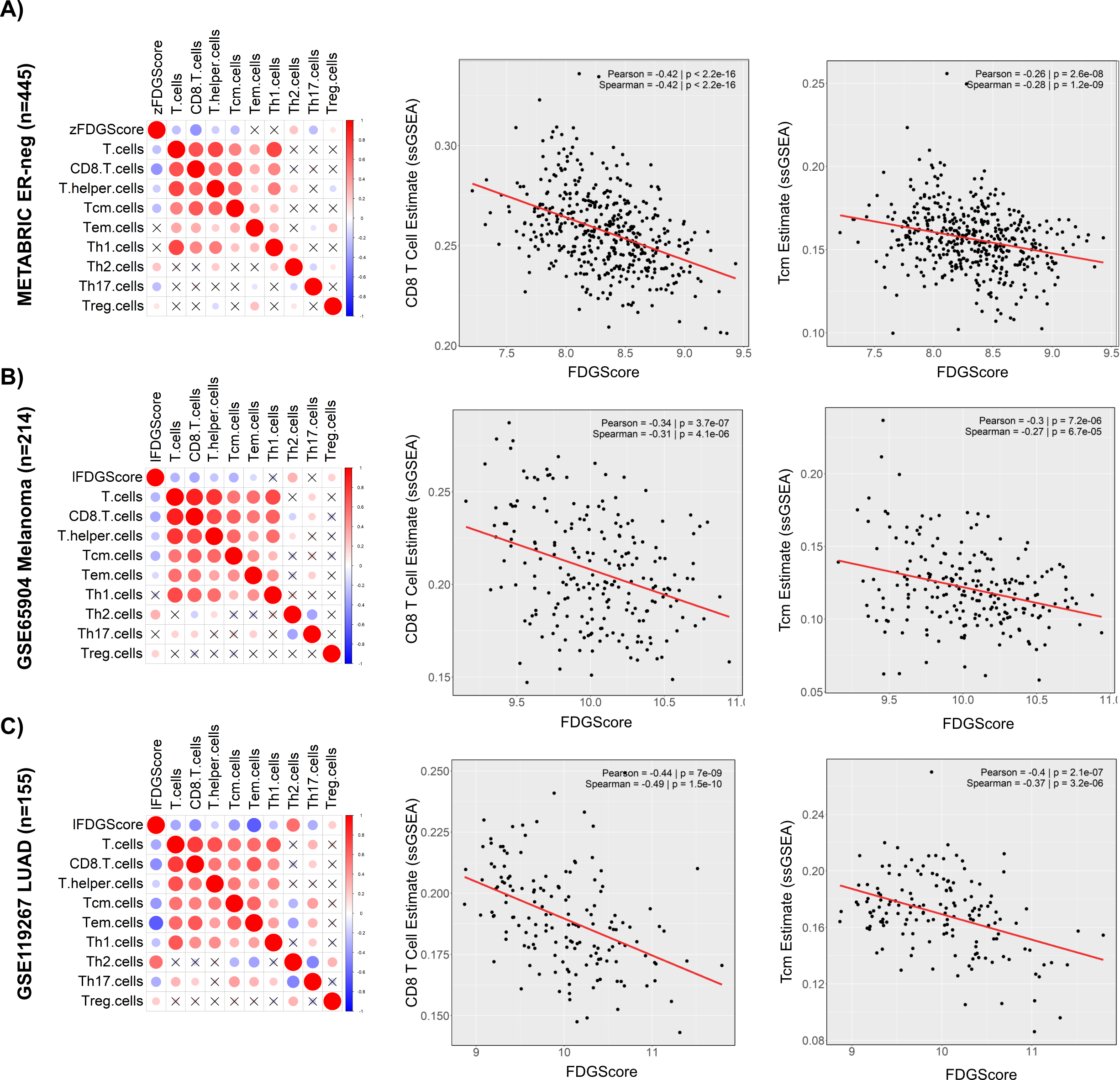

Supplement: Supplementary Figure S1 — Co-expression patterns of glycolysis and immune related genes within individual tumor types. (A, B) RNA expression data was downloaded from TCGA and other datasets (see Methods), and the correlation between expression of selected glycolysis and immune genes was plotted (red = positive correlation; blue = negative correlation. X marks correlation coefficients with p>0.05). Correlation profiles of mRNA expression of selected glycolysis- and immune-related genes across multiple solid tumor types from the TCGA and independent cohorts are shown (A). (B) The expression of specific glycolysis and immune genes was plotted for specific tumor types and the Pearson and Spearman correlation coefficients were calculated. [file DataSheet_1.zip › Supplementary Figures/Supp Figure S5.tiff]

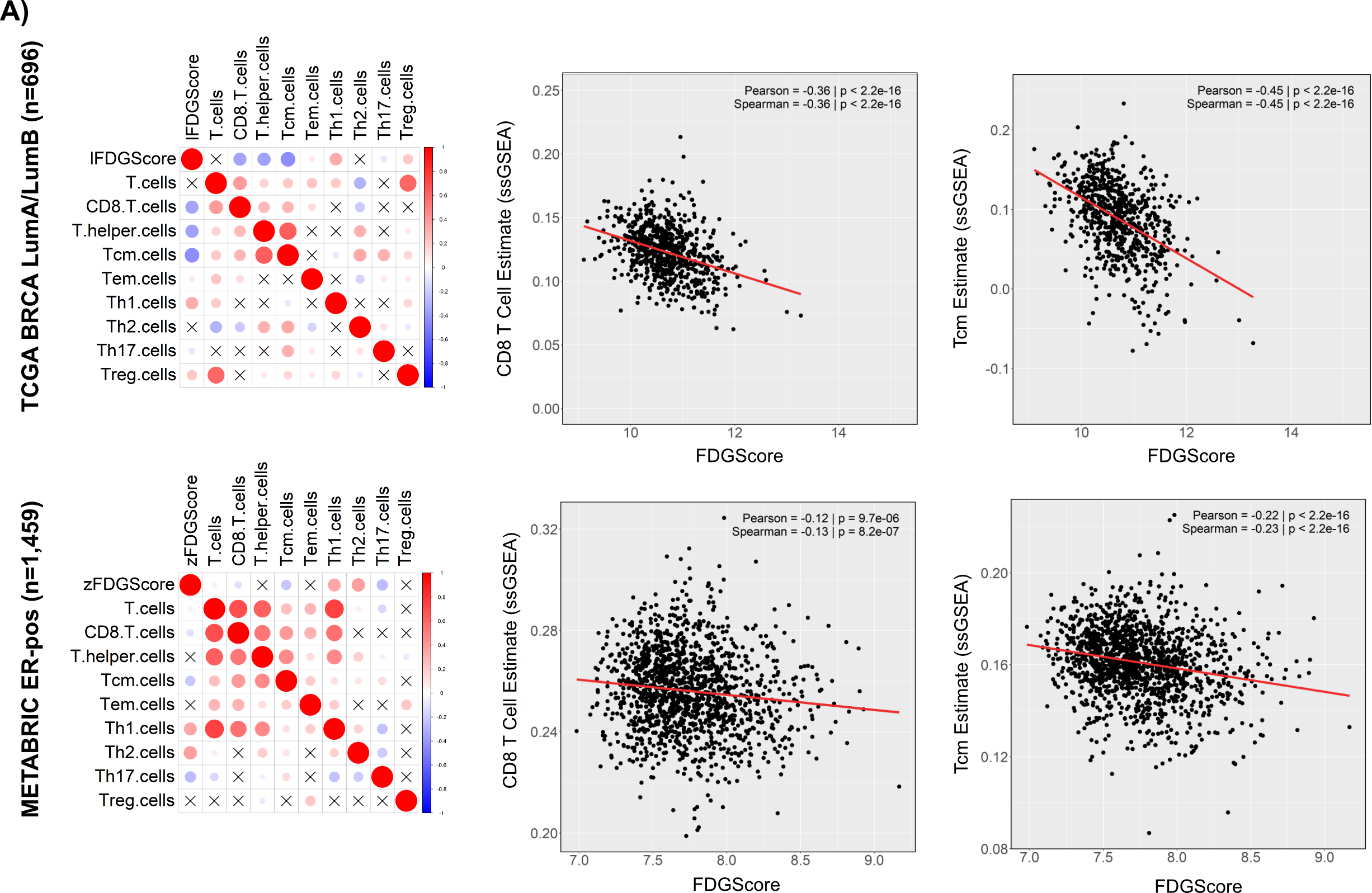

Supplement: Supplementary Figure S1 — Co-expression patterns of glycolysis and immune related genes within individual tumor types. (A, B) RNA expression data was downloaded from TCGA and other datasets (see Methods), and the correlation between expression of selected glycolysis and immune genes was plotted (red = positive correlation; blue = negative correlation. X marks correlation coefficients with p>0.05). Correlation profiles of mRNA expression of selected glycolysis- and immune-related genes across multiple solid tumor types from the TCGA and independent cohorts are shown (A). (B) The expression of specific glycolysis and immune genes was plotted for specific tumor types and the Pearson and Spearman correlation coefficients were calculated. [file DataSheet_1.zip › Supplementary Figures/Supp Figure S6.tiff]

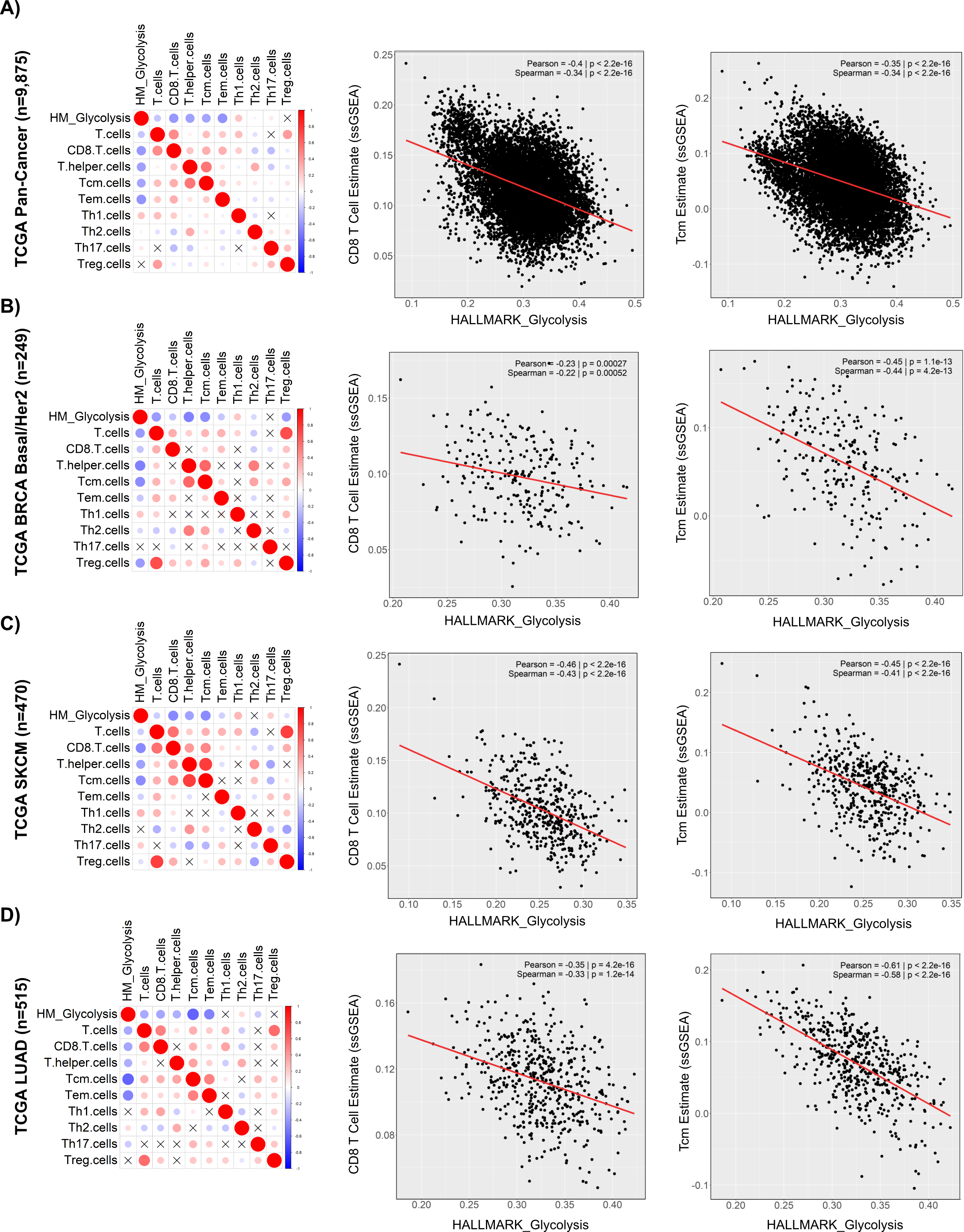

Supplement: Supplementary Figure S1 — Co-expression patterns of glycolysis and immune related genes within individual tumor types. (A, B) RNA expression data was downloaded from TCGA and other datasets (see Methods), and the correlation between expression of selected glycolysis and immune genes was plotted (red = positive correlation; blue = negative correlation. X marks correlation coefficients with p>0.05). Correlation profiles of mRNA expression of selected glycolysis- and immune-related genes across multiple solid tumor types from the TCGA and independent cohorts are shown (A). (B) The expression of specific glycolysis and immune genes was plotted for specific tumor types and the Pearson and Spearman correlation coefficients were calculated. [file DataSheet_1.zip › Supplementary Figures/Supp Figure S7.tiff]

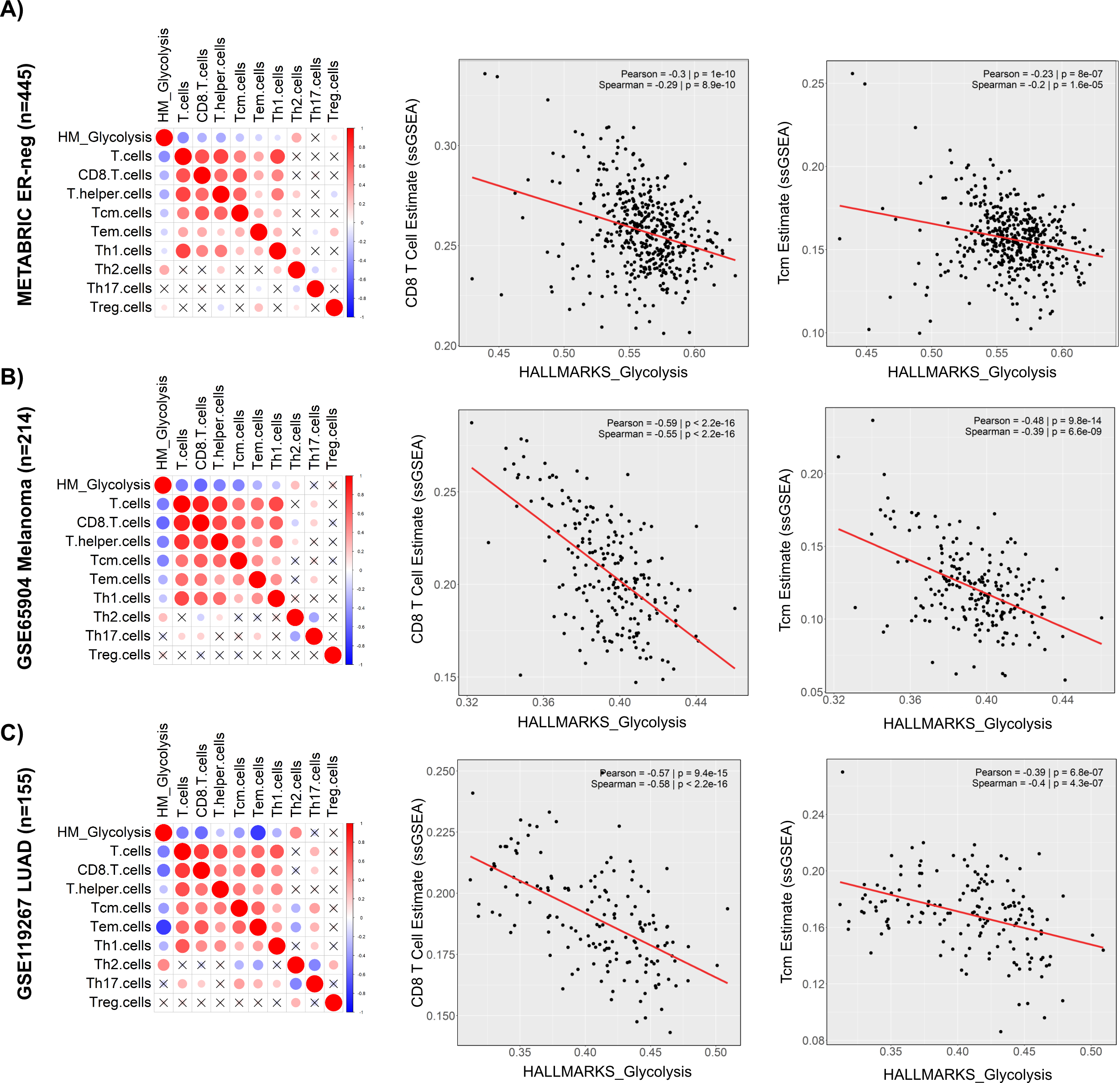

Supplement: Supplementary Figure S1 — Co-expression patterns of glycolysis and immune related genes within individual tumor types. (A, B) RNA expression data was downloaded from TCGA and other datasets (see Methods), and the correlation between expression of selected glycolysis and immune genes was plotted (red = positive correlation; blue = negative correlation. X marks correlation coefficients with p>0.05). Correlation profiles of mRNA expression of selected glycolysis- and immune-related genes across multiple solid tumor types from the TCGA and independent cohorts are shown (A). (B) The expression of specific glycolysis and immune genes was plotted for specific tumor types and the Pearson and Spearman correlation coefficients were calculated. [file DataSheet_1.zip › Supplementary Figures/Supp Figure S8.tiff]

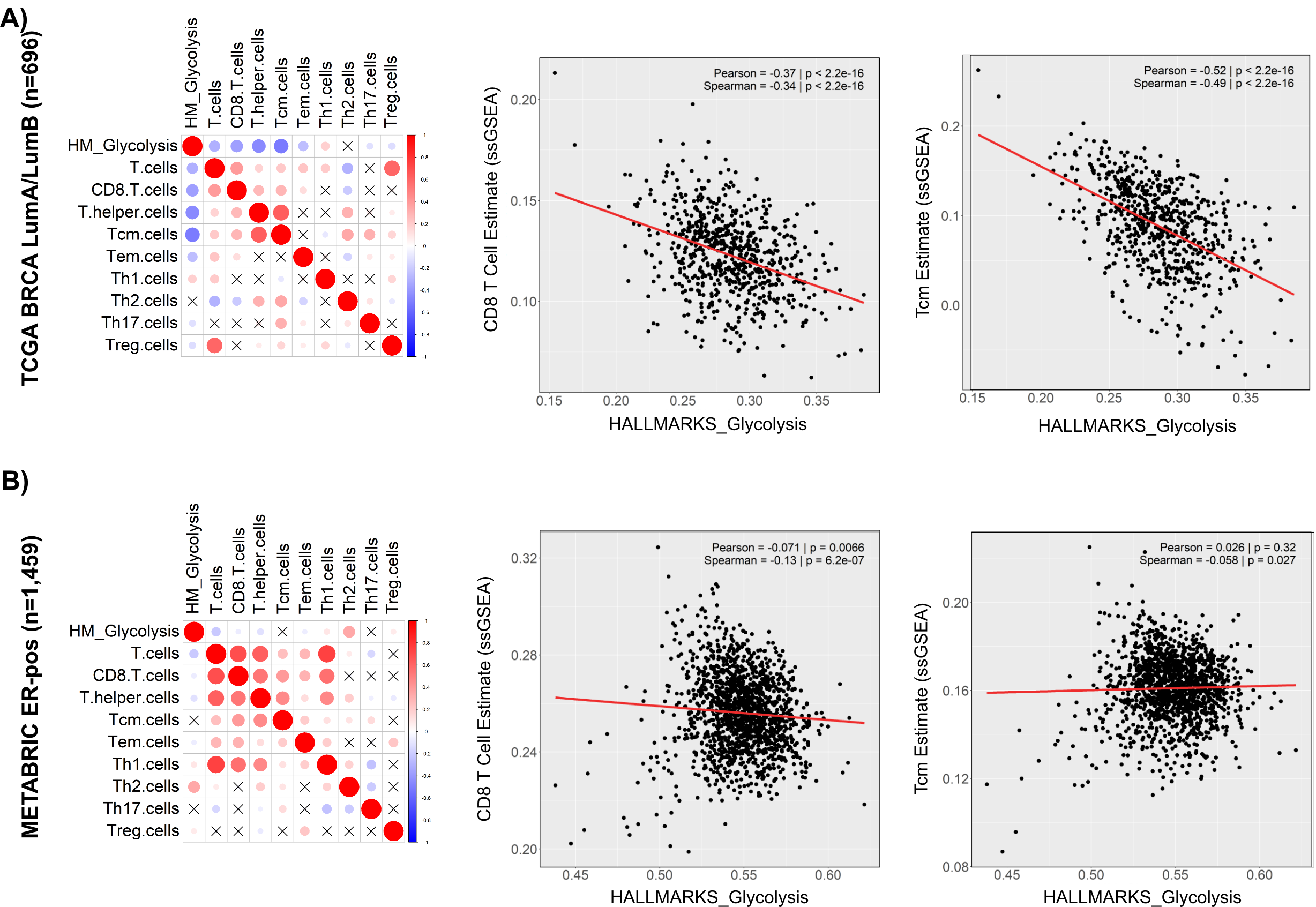

Supplement: Supplementary Figure S1 — Co-expression patterns of glycolysis and immune related genes within individual tumor types. (A, B) RNA expression data was downloaded from TCGA and other datasets (see Methods), and the correlation between expression of selected glycolysis and immune genes was plotted (red = positive correlation; blue = negative correlation. X marks correlation coefficients with p>0.05). Correlation profiles of mRNA expression of selected glycolysis- and immune-related genes across multiple solid tumor types from the TCGA and independent cohorts are shown (A). (B) The expression of specific glycolysis and immune genes was plotted for specific tumor types and the Pearson and Spearman correlation coefficients were calculated. [file DataSheet_1.zip › Supplementary Figures/Supp Figure S9.tiff]
